# Deciphering the molecular signatures of tropical *Areca catechu* L. under cold stress: an integrated physiological and transcriptomic analysis

**DOI:** 10.3389/fpls.2025.1624335

**Published:** 2025-07-22

**Authors:** Han Li, Linbi Zhang, Xinyu Wen, Changlei Ji, Hui Chen, Meng Tian, Fusun Yang, Jun He

**Affiliations:** ^1^ Country School of Tropical Agriculture and Forestry, Hainan University, Haikou, Hainan, China; ^2^ Synthetic Biology Center, School of Future Technology, Fuzhou, Fujian, China; ^3^ Fujian Provincial Key Laboratory of Haixia Applied Plant Systems Biology, Haixia Institute of Science and Technology, College of Life Sciences, Fujian Agriculture and Forestry University, Fuzhou, Fujian, China

**Keywords:** *Areca catechu* L., cold stress, multivariate analysis, RNA-seq, WGCNA

## Abstract

**Introduction:**

*Areca catechu* is a widely cultivated palm species with significant economic and medicinal value. However, *A. catechu* is a tropical plant that is particularly susceptible to low temperatures.

**Methods:**

This study integrates physiological profiling with transcriptomic sequencing to systematically investigate the cold-response mechanisms of *A. catechu*.

**Results:**

Multivariate variance analysis revealed that peroxidase (POD) activity and chlorophyll content are significant biomarkers strongly correlated with cold tolerance. A comprehensive investigation into the temporal expression of genes in response to 24 hours of cold stress was conducted, using RNA-seq analysis. This analysis yielded a substantial number of differentially expressed genes (DEGs), amounting to 20,870, which were found to be subject to temporal regulation. KEGG pathway enrichment analysis revealed substantial activation in three metabolic pathways: phytohormone signaling, alkaloid biosynthesis (tropane/piperidine/pyridine), and flavonoid biosynthesis. The application of Weighted Gene Co-expression Network Analysis (WGCNA), in conjunction with a dynamic tree-cutting algorithm, resulted in the identification of 25 co-expression modules. Eigenvector centrality analysis identified six hub genes responsive to cold stress: *ZMYND15*, *ABHD17B*, *ATL8*, *WNK5*, *XTH3* and *TPS*. The findings of this study delineate three key aspects: (1) temporal dynamics of cold-responsive physiological processes, (2) pathway-level characterization of DEG enrichment patterns, and (3) genetic determinants underlying cold stress adaptation.

**Discussion:**

These findings clarify the time series and core physiological indicators of *A. catechu* during various physiological processes, identify pivotal genes associated with cold stress, and provide a gene-to-phenotype framework for optimizing cold-resilient cultivation protocols and molecular marker-assisted breeding strategies.

## 1 Introduction

The Intergovernmental Panel on Climate Change (IPCC) (www.ipcc.ch, accessed 17 April 2024) has observed in its ‘Climate Change 2024’ report that global temperature instability leads to increased frequency and intensity of heat waves and cold spells. These phenomena can threaten crop yields, especially in areas with minimal climate change impacts. The primary mechanism underpinning this phenomenon involves the fact that extremely low temperatures have the capacity to induce a phase transition in the phospholipids of non-freeze-resistant plant cells. This transition, from a liquid crystalline to a gel phase, results in the destruction of the cell membrane structure, leading to the formation of non-lipid membrane pores on the protoplasmic membrane. These pores, in turn, act as free passageways for numerous electrolytes, ultimately resulting in electrolyte leakage (Ritonga et al., 2021). This process has been shown to result in an increase in malondialdehyde (MDA) content and relative electrical conductivity (REC), as well as structural damage and functional impairment of cellular components (Ding and Yang, 2022; Kidokoro et al., 2022). These effects are manifested through growth retardation, leaf chlorosis and premature floral abscission, which ultimately compromises plant viability (Fei et al., 2021; Wei et al., 2021). Concomitantly, low temperatures disrupt the balance of the ROS system in plants, thereby increasing the activity of antioxidant enzymes such as superoxide dismutase (SOD), peroxidase (POD) and catalase (CAT), which in turn improves cold hardiness (Kosmala et al., 2009; Singh et al., 2016). Therefore, changes in enzyme activities are used to determine whether a plant is subjected to cold stress and to measure the damage caused by the stress to the organism.

In addition to changes at the physiological level, abiotic stress further affects the ability of plants to adapt to the environment and survive by causing transcriptional changes, thereby affecting gene expression (Kidokoro et al., 2022). In *A. catechu*, there is a relative paucity of studies on the molecular mechanisms targeting abiotic stresses. However, genome-wide analysis revealed that drought stress significantly up-regulated the expression of the *A. catechu* bHLH transcription factor family members *AcbHLH22* and *AcbHLH39* (Ali et al., 2024); furthermore, low temperature treatment induced high expression of the *HSP70* gene family member *AcatHSP70-5* (Alam et al., 2024). Notably, further functional studies showed that overexpression of the *AcCHS5* gene of the chalcone synthase gene family was able to reveal a key regulatory module: that is, the transcription factor *AcMYB176* synergized with *AcCHS5* to significantly enhance *A. catechu*’s salinity tolerance by positively regulating flavonoid biosynthesis and enhancing the scavenging capacity of ROS (Jiang et al., 2025). Studies on the mechanisms of transcription factors involved in stress response, including low temperature, are accumulating in the study of oil palm. This palm belongs to the same genus as *A. catechu*. For instance, ectopic expression of the *R2R3-MYB* genes, *EgMYB111* and *EgMYB157*, in oil palm (Elaeis guineensis) was shown to significantly increase the tolerance of plants to a variety of abiotic stresses, including low temperature, by enhancing the activity of antioxidant enzymes and sustaining photosynthetic gas exchange efficiency (Zhou et al., 2022). In the study of cold stress response mechanisms, overexpression of *SlCOR413IM1* under cold stress conditions attenuated cold-induced damage to chloroplast membranes and structures, thereby enhancing cold stress tolerance in tomato (Ma et al., 2018). Conversely, suppression of the *CsBPC2* transcription factor in cucumber (Cucumis sativus) has been shown to elevate electrolyte leakage by 38.7% and malondialdehyde accumulation by 5.2 nmol/g FW, while simultaneously reducing key antioxidant enzyme activities (Meng et al., 2023). In Arabidopsis, *ICE1* acts as a major regulator of *CBF*, interacting with protein kinase 3 (*MPK3*) and *MPK6* to negatively regulate *CBF* expression in plants and plant freezing tolerance (Li et al., 2017). In summary, this series of reactions constitutes an adaptive compensatory mechanism for maintaining cellular homeostasis and physiological function in plants under cold stress.

As a tropical cash crop, *A. catechu* produces medicinal phytochemicals in its seeds, rind, and flower buds (Peng et al., 2015), with demonstrated bioactivities including digestive enhancement, neuro-cardiovascular modulation, and broad-spectrum antioxidant/anti-inflammatory effects (Ghate et al., 2014; Gilani et al., 2004; Hamsar et al., 2011; Holdsworth et al., 1998). As the primary cultivation zone for *A. catechu*, Hainan Province’s tropical monsoon climate, marked by pronounced dry-wet seasonality, has biased abiotic stress research toward drought adaptation, overlooking cold stress impacts. Because Hainan Province is surrounded by the sea on all sides, it makes it more vulnerable to unstable cold snaps. Based on the statistical analysis of temperature data from May 1, 2022, to April 29, 2023, in four regions of Hainan Province, east, west, south, and north, the average daily temperature in Hainan Province ranged from 12.7 to 32.1°C, with a daily minimum temperature of 6.8°C. The low temperature period was mainly concentrated in December and January, during which the days with an average daily temperature lower than 15°C ranged from 15 to 35, and the average duration of consecutive low temperatures was 5 to 10 days (**Supplementary Figure S1**). The optimum growing temperature of *A. catechu* is 20-25°C. When the temperature is lower than 16°C, the leaf tips will be damaged, lower than 10°C, the leaves will turn yellow, and lower than 5°C, it will be subjected to severe cold damage, which further threatens the yield of *A. catechu* (Qin and Fan, 2010). As a tropical economic forest tree, *A. catechu* is often neglected due to its low-temperature sensitivity. The molecular basis of cold acclimation in *A. catechu* remains poorly characterized, with critical gaps in understanding physiological adaptation thresholds and transcriptional regulators of stress-responsive pathways.

To delineate the cold-response mechanisms of *A. catechu*, comparative physiological profiling was performed between control (26°C) and cold-stressed (10°C) plants, employing multivariate statistical methods to identify critical biomarkers. Subsequently, RNA-Seq was employed to analyses the DEGs and the enrichment pathways of differentially expressed genes in *A. catechu* leaves under different durations of cold stress. The identification of hub genes was accomplished by constructing a co-expression network employing weighted gene co-expression network analysis (WGCNA). The upstream regulatory genes associated with cold stress in *A. catechu* were verified using reverse transcription (RT)-quantitative real-time PCR (RT-qPCR). The present study aims to analyses the molecular characteristics of cold stress in *A. catechu*, thereby providing a theoretical basis for cold-resistant cultivation and variety selection of *A. catechu*.

## 2 Materials and methods

### 2.1 Plant material and low temperature stress treatment

Two-leaf stage ‘Reyan No.1’ *A. catechu* seedlings exhibiting uniform growth vigor were selected as experimental materials. The treatment group underwent controlled cold stress acclimation in a ZRG-250A-L phytotron (Shanghai Binglin Technology Company, China), regulated at 10°C; while an LED illumination system maintained photosynthetic photon flux density (PPFD) at 1200 μmol·m^-2^·s^-1^; relative humidity: 70% RH. A 12-hours photoperiod (08:00-20:00 hours) was implemented through programmable dimming protocols, establishing precise daily illumination cycles. Seedlings cultivated under ambient temperature conditions (26 ± 1°C) with identical light and humidity parameters served as the control group. All specimens completed 72-hours preconditioning at target irradiance and humidity setpoints before experimental onset. The penultimate fully expanded leaves from *A. catechu* were systematically examined during days 2, 4, 6, 8, and 10 post-cold exposure (C2d, C4d, C6d, C8d, and C10d, respectively). Sampling was conducted synchronously between 09:00 and 10:30 AM. Five mixed samples were taken at each time point, immediately frozen in liquid nitrogen, and stored at -80°C for physiological index analysis.

**Figure 1:**
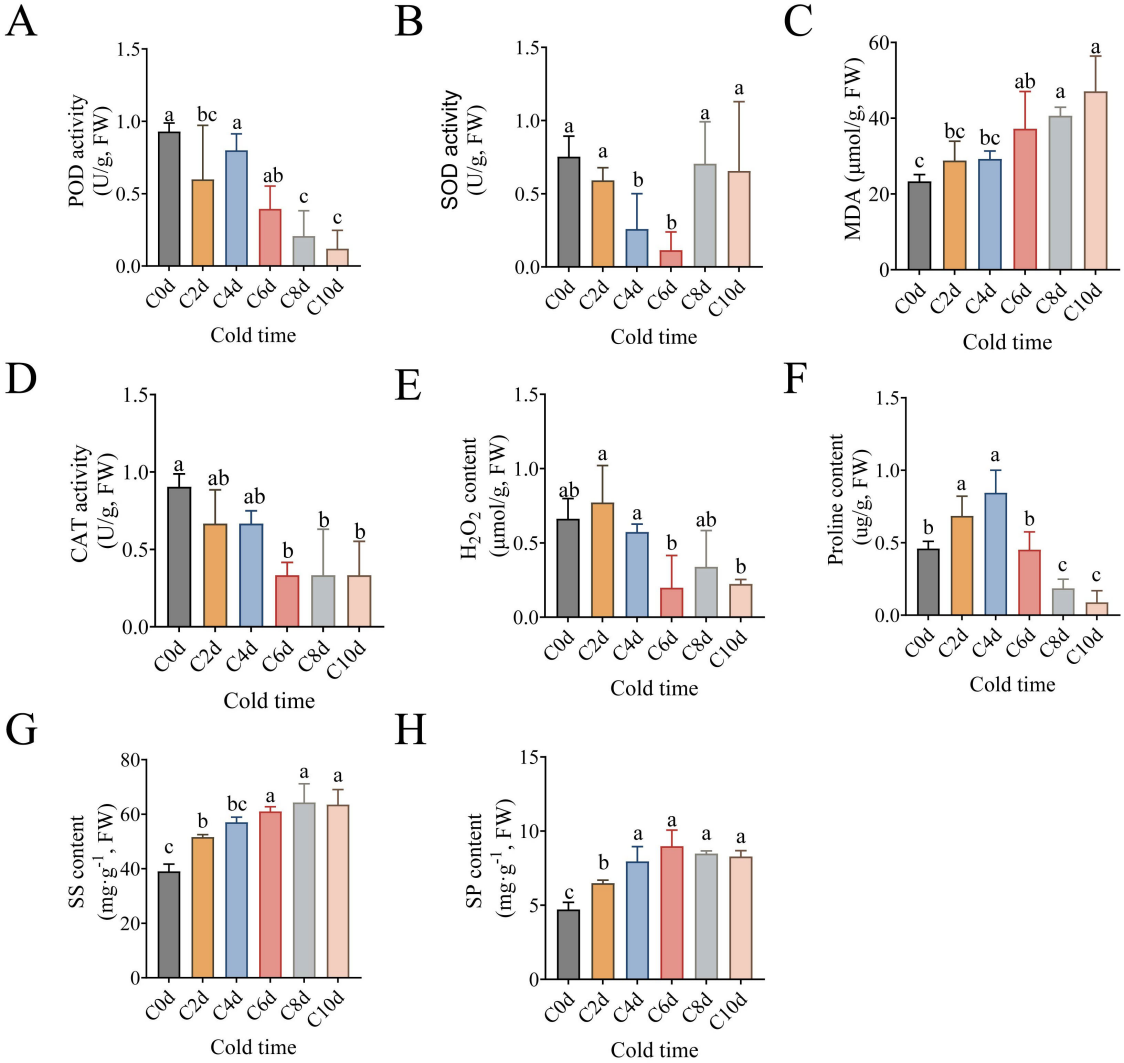
Physiological response of *A catechu* to cold stress. **(A)** Peroxidase (POD). **(B)** superoxide dismutase (SOD). **(C)** Malondialdehyde (MDA). **(D)** Catalase (CAT). **(E)** Hydrogen peroxide (H_2_O_2_). **(F)** Free Proline. **(G)** Soluble sugar (SS). **(H)** Soluble protein (SP). C0d denotes *A. catechu* grown at 26°C (control), where Figures **(A-H)** Data were analyzed using Tukey’s method of multiple comparisons test at a significance level of P < 0.05. Lowercase letters (a, b, c) indicate statistically homogeneous groups based on Tukey’s multiple comparison test (P<0.05). Groups sharing the same letter are not significantly different, while those with different letters show significant differences.

Following the measurement and analysis of the physiological indices of *A. catechu*, it was determined that the subject exhibited significant physiological changes within two days of exposure to cold stress. Specifically, the antioxidant enzyme-related indices POD and CAT decreased significantly (P < 0.05) by 59.29% and 36.07% respectively, exposure (10°C) within the 48-hour period (**Figures 1A, D**). Consequently, cold samples at 0, 6 h, 8 h, and 24 h were utilized to compare the transcriptional profiles. Each group comprised three biological replicates, with individual replicates consisting of leaves collected from five randomly selected seedlings to minimize microenvironmental differences. This study aims to determine the physiological response of *A. catechu* to cold stress.

### 2.2 Determination of physiological response to cold stress in *A. catechu*

Chlorophyll content was determined spectrophotometrically following Wang (Wang, 1900). Absorbance was measured at 663 nm (pigment a), 645 nm (pigment b), and 470 nm (carotenoids) using quartz cuvettes (path length 1 cm) in a T400F UV-Vis spectrophotometer (Beijing Pudian General Instrument Co., Ltd.). Pigment concentrations (mg/L) were calculated as follows: Chlorophyll a = 12.21*A663 - 2.81*A645 and Chlorophyll = 20.13*A645 - 5.03*A663.

Relative water content (RWC) was determined through gravimetric measurements (Du et al, 2018). Freshly harvested leaves were blotted to remove surface moisture and immediately weighed to obtain fresh weight (FW). Samples were then fully immersed in distilled water within sealed containers and incubated in the dark at room temperature until fully saturated. Saturation was confirmed by repeated weighing at 2–4 hours intervals, with the plateau mass recorded as turgid weight (TW). Subsequently, samples were oven-dried at 75°C until constant mass was achieved (verified by repeated weighing after 2–4 hours intervals during drying), cooled in a desiccator, and weighed to determine dry weight (DW). RWC (%) was calculated using the formula: [(FW - DW)/(TW - DW)] × 100.

Leaf relative electrical conductivity (REC) was quantified using a conductometric method adapted from Li (Li, 2000). Fresh leaf samples were immersed in 10 mL ultrapure deionized water, with initial conductivity (S_0_) measured using a DDS-11A conductivity meter (Shanghai Lei magnet Instruments Co., Ltd.). Intermediate conductivity (S_1_) was determined after 60 min at 20.0°C in a constant-temperature water bath. Samples were boiled in sealed test tubes for 30 min, cooled to 20.0°C with continuous shaking in a thermostatic water bath, and final conductivity (S_2_) was recorded. Relative electrolyte leakage (%) was calculated as: (S_1_-S_0_)/(S_2_-S_0_).

Free proline levels were assayed via ninhydrin reaction using L-proline calibration standards, expressed as μg/g FW (Patel and Vora, 1985). Fresh *A. catechu* leaves (0.1 g) were homogenized in 3% sulfosalicylic acid. The homogenate was centrifuged (12,000 g, 15 min, 4°C). The supernatant (500 μL) was reacted with acid ninhydrin reagent (500 μL) and glacial acetic acid (500 μL) at 100°C for 60 min. After quenching on ice, it was extracted with toluene (1 mL) and centrifuged (3,000 g, 5 min). The absorbance was then measured at 520 nm; Malondialdehyde (MDA) concentration was quantified through the thiobarbituric acid reactive substances assay with modifications from Wu (Wu et al., 2020). Fresh leaves (0.1 g) were homogenized in 5 ml of a mixture of 0.25% thiobarbituric acid (TBA) and 10% trichloroacetic acid (TCA). The homogenates were incubated in sealed test tubes at 95°C for 45 min and then immediately quenched on ice. After centrifugation (12,000 g, 15 min, 4°C), supernatant absorbance was measured at 532 nm and 600 nm; Soluble protein (SP) concentration was determined using the Bradford method (Jones et al., 1989). After centrifugation (12,000 × g, 10 minutes, 4°C) to clarify the sample, 5–20 μL of the supernatant was mixed with 250 μL of Bradford reagent (Coomassie Brilliant Blue G-250). After incubation at room temperature for 10 minutes, the absorbance was measured at 595 nm using a spectrophotometer. A standard curve was prepared using bovine serum albumin (BSA; 0–2000 μg/mL) in the extraction buffer. Protein concentration was calculated by linear regression against the standard curve. Soluble sugars (SS) were assayed by the anthrone-H_2_SO_4_ method (Khattak et al., 2022). Ethanol extracts (0.2 mL) were mixed with ice-cold anthrone reagent (0.2% in H_2_SO_4_), heated (100°C, 10 min), cooled, and read at 620 nm. Values were derived from a glucose standard curve. H_2_O_2_ content was determined using a commercial detection kit (Solarbio, BC3590, China).

### 2.3 Determination of net photosynthetic rate

Photosynthetic parameter quantification was conducted on the penultimate fully expanded leaves of *A. catechu* using an LI-6800 Portable Photosynthesis System (Li-Cor Biosciences, USA). During the experimental period, measurements occurred daily between 08:30 and 11:30 to capture peak photosynthetic activity. Before data logging, leaves were acclimatized within the chamber for 30 min under target conditions to achieve steady-state gas exchange. For each sample, 10 consecutive readings (30 seconds apart) are required to ensure data stability. Environmental control parameters were strictly maintained as follows: leaf chamber temperature maintained at 26°C (± 1°C), PPFD of 1200 μmol·m^-2^·s^-1^, ambient CO_2_ concentration (400 ± 5 μmol·mol^-^¹), and relative humidity (RH) controlled at 50-75%.

### 2.4 *A. catechu* peroxidase activity under cold stress

The measurement of CAT activity, POD activity and SOD activity was conducted in accordance with the manufacturer’s instructions, employing the catalase (CAT) activity assay kit (Solarbio R BC0205), the peroxidase (POD) assay kit (Solarbio R BC0090) and the superoxide dismutase (SOD) assay kit (Solarbio R BC0175).

### 2.5 RNA extraction, cDNA library preparation, and sequencing

The penultimate leaves of *A. catechu* (0.2 g) were harvested without RNase and quickly snap-frozen in liquid N_2_. The total RNA extraction process was conducted in strict accordance with the protocol stipulated in the RNA kit provided by Beijing Tiangen Biotechnology Co., Ltd (Beijing, China). Following this, the extracted total RNA was transferred on dry ice to Shanghai Major Biomedical Technology Co., Ltd (Shanghai, China) for on-board sequencing. RNA integrity was rigorously validated through 1.5% agarose gel electrophoresis (sharp 28S/18S bands), Nanodrop 2000 quantification (A_260_/A_280_ ≥ 1.95; A_260_/A_230_ > 2.0), and Agilent 5300 Bioanalyzer analysis (RIN ≥ 8.0). Polyadenylated mRNA was isolated from 1 μg total RNA via oligo(dT)_25_ magnetic bead capture (65°C, 15 min binding) with three stringent wash cycles.

mRNA was fragmented to 250–300 nt using Mg²^+^ buffer (94°C, 7 min). First-strand cDNA synthesis employed random hexamers and ProtoScript II RT (42°C, 50 min). End repair (20°C, 30 min), dA-tailing (37°C, 30 min), and TruSeq adapter ligation (20°C, 15 min) were sequentially performed. Size selection (SPRIselect beads) targeted 350 ± 15 bp inserts and index incorporation via PCR (12 cycles). Validated libraries (Qubit/Agilent DV_200_ > 0.8) underwent 150 bp paired-end sequencing on NovaSeq X Plus (Illumina), yielding >20M reads/sample at Q30 ≥ 90%.

### 2.6 Differentially expressed gene identification

Differential expression analysis was performed using DESeq2 with raw read counts (Rapaport et al., 2013). Meanwhile, transcript abundance was quantified as FPKM (Fragments Per Kilobase per Million mapped reads). Significantly differentially expressed genes (DEGs) were identified through concurrent thresholds: variable importance in projection (VIP) > 1.0 derived from partial least squares-discriminant analysis (PLS-DA), false discovery rate (FDR) < 0.05 (Benjamini-Hochberg adjusted), and absolute log_2_ fold change ≥ 1 (|FC| ≥ 1). Functional enrichment analysis included Gene Ontology (GO) terms using Goathoods (Version 0.6.5) and semantic similarity reduction, and KEGG pathways via KOBAS employing hypergeometric tests (FDR < 0.05) validated by pathway topology analysis (Li et al., 2023).

### 2.7 Weighted gene co-expression network analysis

RNA-seq data for Weighted Gene Co-expression Network Analysis (WGCNA) were obtained from 12 samples across four cold stress time points. Data preprocessing was performed using RSEM (v1.3.1), retaining transcripts with an expression mean ≥1 and coefficient of variation ≥0.1. Subsequent WGCNA of cold stress-responsive differentially expressed genes in *A. catechu* was implemented using the R package (v3.4.1) (Langfelder and Horvath, 2008). Module identification parameters included: a signed weighted network, soft-thresholding power (β) = 9, minimum module size = 30, minimum module membership (kME) threshold = 0.3, and a merge cut height of 0.25. Module-trait associations were evaluated using Spearman correlation analysis.

### 2.8 Quantitative real-time PCR

Quantitative PCR was performed on a real-time Qubit 4.0 (thermo, USA). Each 20 μL reaction mixture contained 10 μL of 2 × TransStart Top Green qPCR SuperMix (TransGen Biotech, Beijing, China), 0.2 μM of each primer (sequences listed in **Supplementary Table S1**), and 1 μL of diluted cDNA template. The thermal cycling conditions consisted of an initial denaturation at 95°C for 30 s, followed by 40 cycles of 95°C for 5 s and 60°C for 30 s, with a final melt curve analysis step (65°C to 95°C, increment 0.5°C per 5 s) to verify amplicon specificity. Gene expression levels were calculated using a relative quantification method (2^-ΔΔCT)^) with *β-actin* as the internal reference gene (Li et al., 2021).

### 2.9 Processing and analyzing data

Statistical analyses were conducted in SPSS 20.0 (IBM Corp.). After validating parametric assumptions, a one-way ANOVA with Tukey’s *post hoc* test (P < 0.05) was utilized to conduct group comparisons. The data visualization process involved the utilization of GraphPad Prism 10.1.2 and OriginPro 9.8.0 software.

## 3 Result

### 3.1 Changes in leaf phenotypes of *A. catechu* at low temperatures

**Figure 2:**
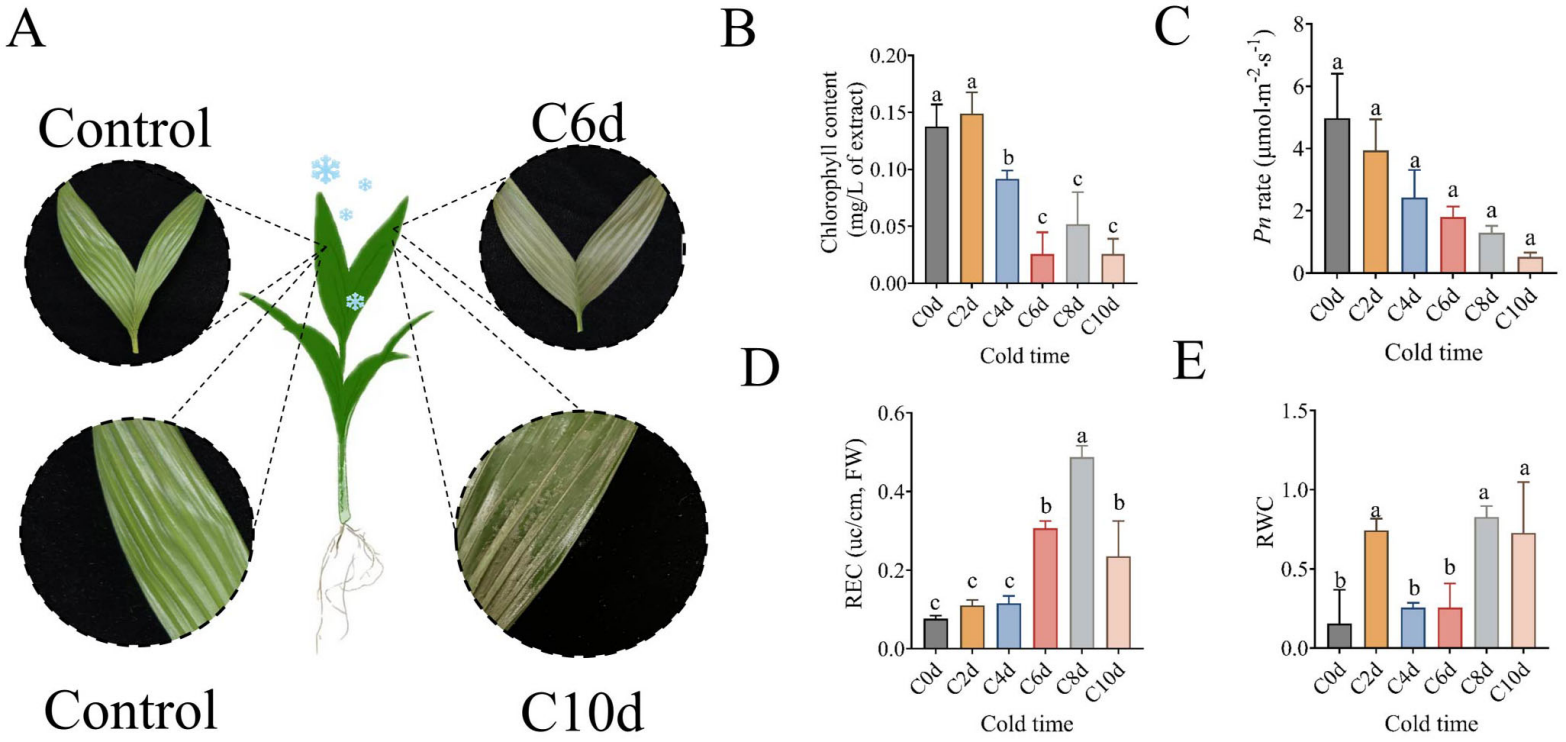
Phenotypic changes in leaves of *A. catechu* after low temperature stress and analysis of data related to phenotype. **(A)** Phenotypic changes in the leaves of *A. catechu* at 6 days and 10 days after cold stress. **(B)** Changes in chlorophyll content of *A. catechu* leaves. **(C)** Changes in the net photosynthetic rate of *A. catechu*. **(D)** Changes in leaf relative conductivity **(E)** Changes in relative leaf water content. C0d (0 days) indicates *A. catechu* grown at 26°C (control). Data were analyzed using Tukey’s method of multiple comparison test at a significance level of P < 0.05. Lowercase letters (a, b, c) indicate statistically homogeneous groups based on Tukey’s multiple comparison test (P<0.05). Groups sharing the same letter are not significantly different, while those with different letters show significant differences.

It has been demonstrated that plant leaves are capable of rapidly sensing and responding to changes in temperature. Following a period of six days during which the temperature was maintained at a low level, a significant loss of green coloration was observed in the leaves of *A. catechu* (**Figure 2A**). Concurrently, the chlorophyll content exhibited a 17.07% decrease compared to the 0 days reference point (**Figure 2B**), and the net photosynthetic rate of the leaves demonstrated a 63.90% reduction (**Figure 2C**). Following a period of ten days during which the leaves were exposed to low temperatures, the leaves exhibited significant signs of wilting, accompanied by an increase in pigmentation and the detachment of the superficial layer. This process ultimately resulted in a distinctive water-soaked morphology, as illustrated in **Figure 2A**. Concurrently, the chlorophyll content and net photosynthetic rate attained their minimum values, while the leaf relative water content (RWC) exhibited a 1.06-fold increase in comparison with the 0 days measurements (**Figure 2E**). The relative electrical conductivity (REC) of *A. catechu* leaves was the subject of further analysis, which revealed a sharp increase from 4 to 8 days of stress, followed by a rapid decrease after 8 days (**Figure 2D**).

### 3.2 Physiological response of *A. catechu* seedlings to low temperature

To systematically evaluate the cold stress-induced physiological adaptations in *A. catechu* leaves, the following metrics were quantified: key antioxidant enzyme activities (POD, SOD, CAT), oxidative stress markers (H_2_O_2_ and MDA), and osmoregulatory metabolites (proline, soluble protein, and soluble sugar) (**Figure 1**). Following a low-temperature treatment, *A. catechu* leaves exhibited distinct antioxidant response patterns: POD activity demonstrated a precipitous decline of 55.1% within 2 days to 0 days, while SOD activity exhibited progressive attenuation throughout the initial 6 days of stress (**Figure 1B**). During the late stress phase (days 8–10), the activities of POD and CAT on days 8 and 10 were reduced by an average of 73.30% and 40.95%, respectively, compared with the activity of SOD (**Supplementary Figure S3**). CAT activity and H_2_O_2_ accumulation notably showed synchronized temporal patterns, decreasing with prolonged stress duration (**Figures 1D, E**).

The temporal pattern of cold induction of osmoregulatory metabolites was observed to differ. A marked elevation in MDA of 50.5% was observed at 48 hours post-treatment in comparison with untreated controls (C0d) (**Figure 1C**). Proline (Pro) exhibited a transient accumulation pattern, peaking at 4 days before declining below initial levels by day 6 (**Figure 1F**). Conversely, soluble sugars (SS) exhibited a sustained augmentation, reaching maximal levels (39.3% above controls) at 8 days (**Figure 1G**). Conversely, soluble proteins (SP) exhibited an earlier peak at 6 days, with a 47.6% increase observed in comparison to the control group (**Figure 1H**).

**Figure 3:**
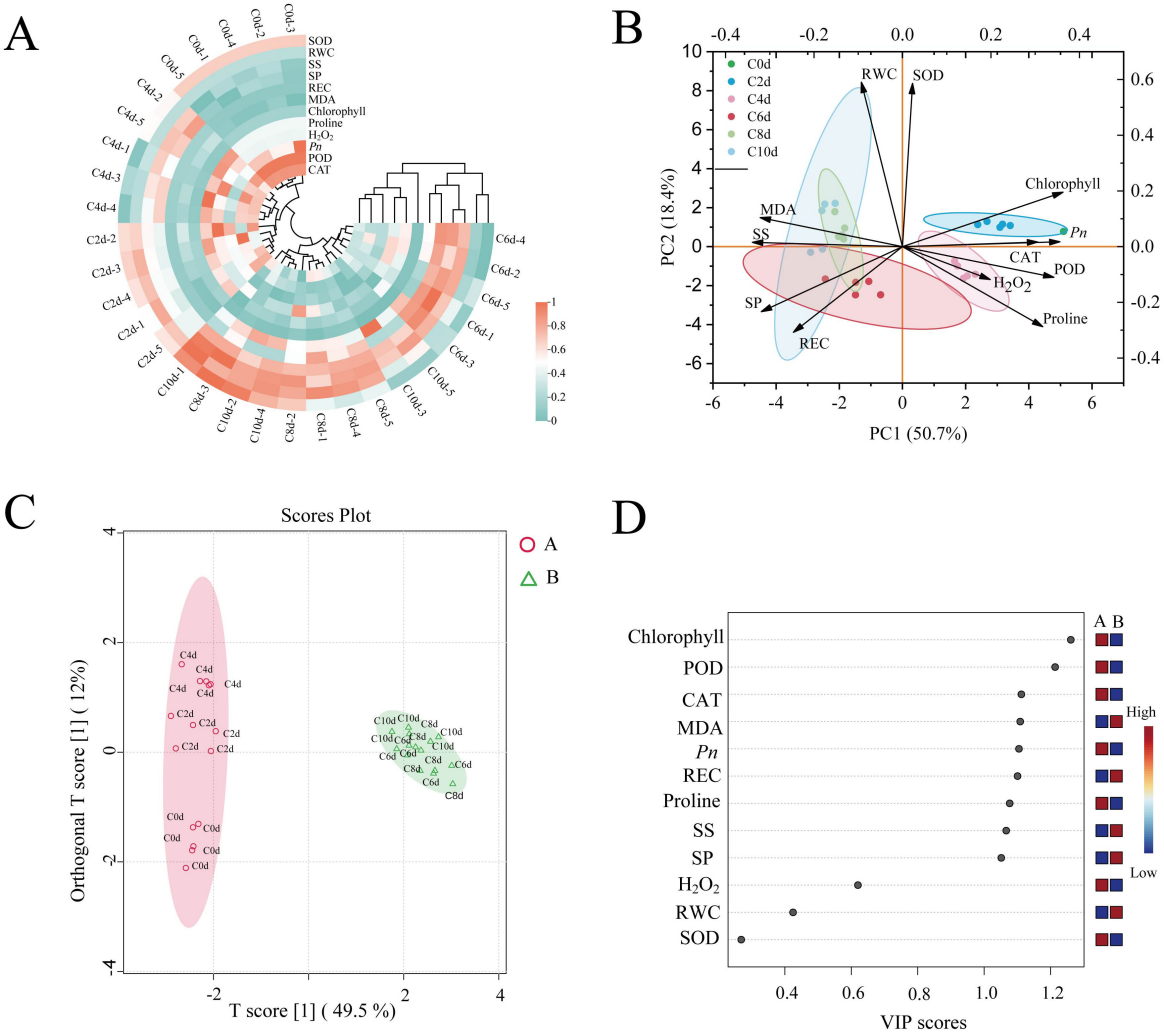
Multivariate analysis of physiological indices of *A. catechu*. **(A)** Clustering heat map of physiological indicators of *A. catechu*. **(B)** Principal component analysis (PCA) of physiological indices of *A. catechu*. **(C)** Orthogonal partial least squares discriminant analysis (OPLS-DA) scatterplots. **(D)** Orthogonal partial least squares discriminant analysis (OPLS-DA) VIP scores.

Twelve physiological indices of *A. catechu* were subjected to a distribution analysis (**Supplementary Figure S2**), and the clustering of physiological parameters of *A. catechu* under cold stress was analyzed (**Figure 3A**). The physiological profile of *A. catechu* under cold stress was systematically characterized through the evaluation of normality for twelve biochemical parameters. Multivariate analysis categorized the samples that had undergone cold treatment into two distinct temporal clusters: early-phase responses (C0d-C4d, 0–4 days) and late-phase adaptations (C6d-C10d, 6–10 days). This result is consistent with the orthogonal partial least squares discriminant analysis (OPLS-DA) differentiation pattern (**Figure 3C**). The results of the principal component analysis (PCA) demonstrated a total variability of 50.7% for PC1 (see **Figure 3B**). The SOD, POD and CAT, Chlorophyll, H_2_O_2_ and Proline were found to be positively loaded, while RWC and REC, MDA, SS and SP contents were found to be negatively loaded. To further assess the key physiological indicators of *A. catechu*, OPLS-DA was employed, and a VIP threshold value greater than 1.0 was designated as the screening criterion for these key physiological indicators. The results indicated that the VIPs of Chlorophyll, POD, CAT, MDA, REC, Pro, SS, and SP were all greater than 1. However, it was observed that only the VIPs of POD (VIP = 1.214) and Chlorophyll (VIP = 1.261) had VIP values greater than 1.2. This finding indicates that POD and chlorophyll are pivotal physiological parameters for assessing cold tolerance in *A. catechu* (**Figure 3D**; **Supplementary Table S2**).

### 3.3 RNA sequencing of *A. catechu* under low temperature

**Figure 4:**
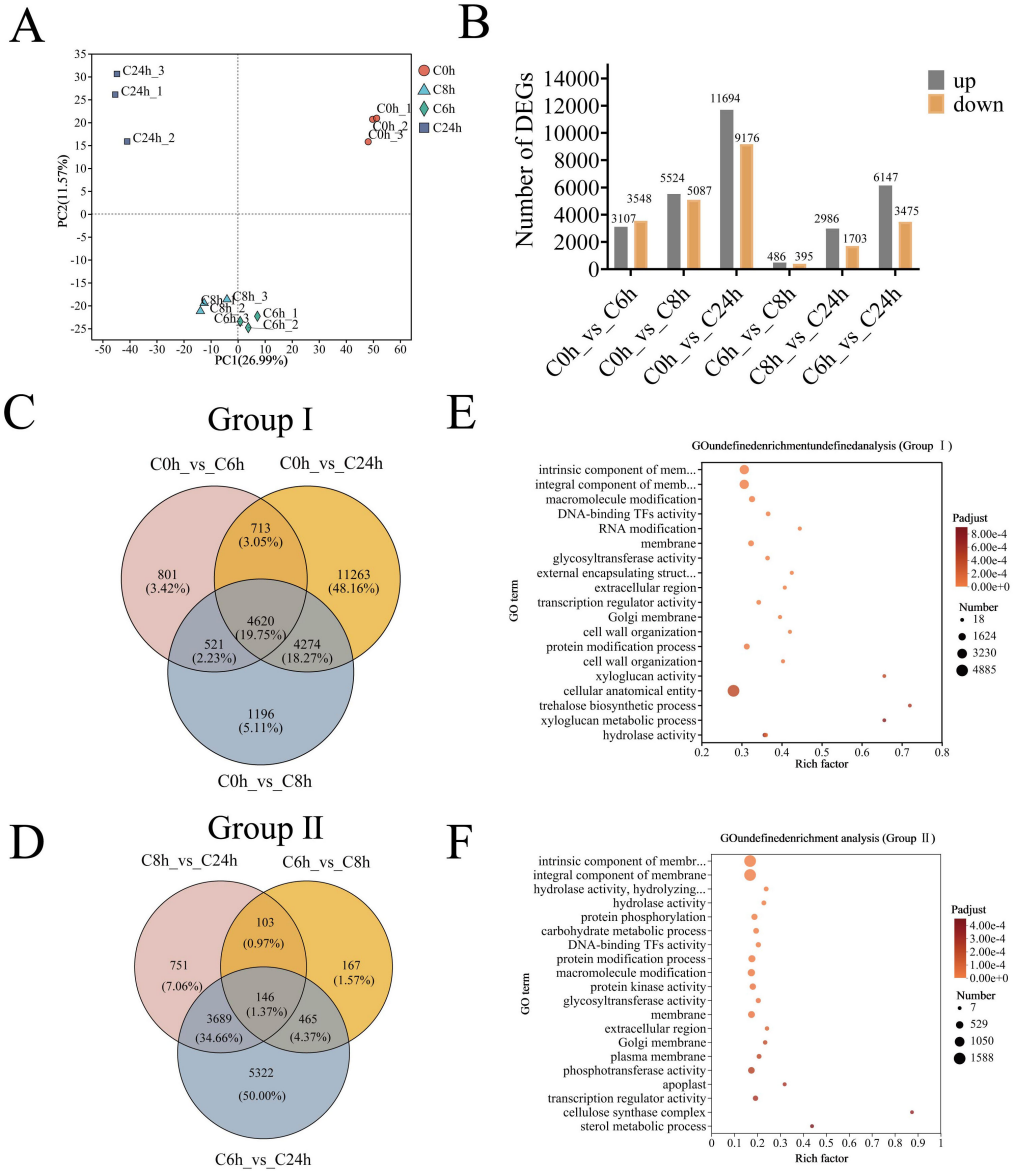
Identification and GO enrichment analysis of differentially expressed genes in *A. catechu* after cold stress. **(A)** Inter-sample principal component analysis. **(B)** DEGs for two-by-two comparisons between samples at different time points. **(C)** Venn diagram of group I DEGs. **(D)** Venn diagram of group II DEGs. **(E)** GO enrichment analysis of DEGs in group I **(F)** GO enrichment analysis of DEGs in group II.

The Phenotypic and physiological responses of *A. catechu* to cold stress were the basis for collecting leaves for transcriptome sequencing at 0, 6, 8, and 24 hours after cold stress. The sequencing library yielded between 41,153,456 and 48,082,506 high-quality reads from 12 A*. catechu* samples, with a minimum of 97.85% Q20 bases and 93.42% Q30 bases, and an average GC content of 45.93% (**Supplementary Table S3**). The clean data of all samples were assembled from scratch using Trinity, and the assembly results were evaluated for optimization. The total number of unigenes obtained from the assembly process was 125,046 (**Supplementary Table S4**). After this, the clean reads of each sample were compared with the reference sequences obtained from Trinity assembly, and the mapping rates ranged from 85.03% to 83.32% (**Supplementary Table S5**). Furthermore, PCA of the 12 samples exhibited a high degree of concordance between the three biological replicates, with significant differences between groups (**Figure 4A**). Consequently, the transcriptomic data from this study can be utilized for further analysis.

### 3.4 Differential gene expression analysis

To elucidate the molecular response mechanism of *A. catechu* to low-temperature stress, two comparison groups were established, and six distinct comparisons were analyzed at four time points (C0 h vs. C6 h, C0 h vs. C8 h, and C0 h vs. C24h, C6 h vs. C8 h, C6 h vs. C24h, and C8 h vs. C24h). The identification of DEGs was conducted employing stringent thresholds (adjusted P < 0.05 and |log2FC| ≥ 1), which resulted in the identification of a total of 533,286 DEGs (**Figure 4B**). Subsequently, an analysis was conducted of the significantly DEGs between different stress time nodes in comparison to the control. The C0h-C6h comparison yielded 6,655 DEGs (3,107 up-/3,548 down-regulated), followed by 10,611 DEGs (5,524/5,087) at C0h-C8h, which culminated in 20,870 DEGs (11,694/9,176) at C0h-C24h (**Figure 4B**; **Supplementary Table S6**). Furthermore, a total of 4620 and 146 shared differentially expressed genes (DEGs) were identified in the two comparison groups (**Figures 4C, D**).

The application of functional annotation of DEGs through Gene Ontology (GO) enrichment revealed distinct functional partitioning. Group I exhibited significant enrichment in cellular components, encompassing the plasma membrane (along with its intrinsic components) and cellular structures (**Figure 4E**). In contrast, Group II exhibited expanded functional diversity, with additional enrichment in biological processes (protein modification) and molecular functions (phosphotransferase activity), while maintaining membrane-related components (**Figure 4F**).

**Figure 5:**
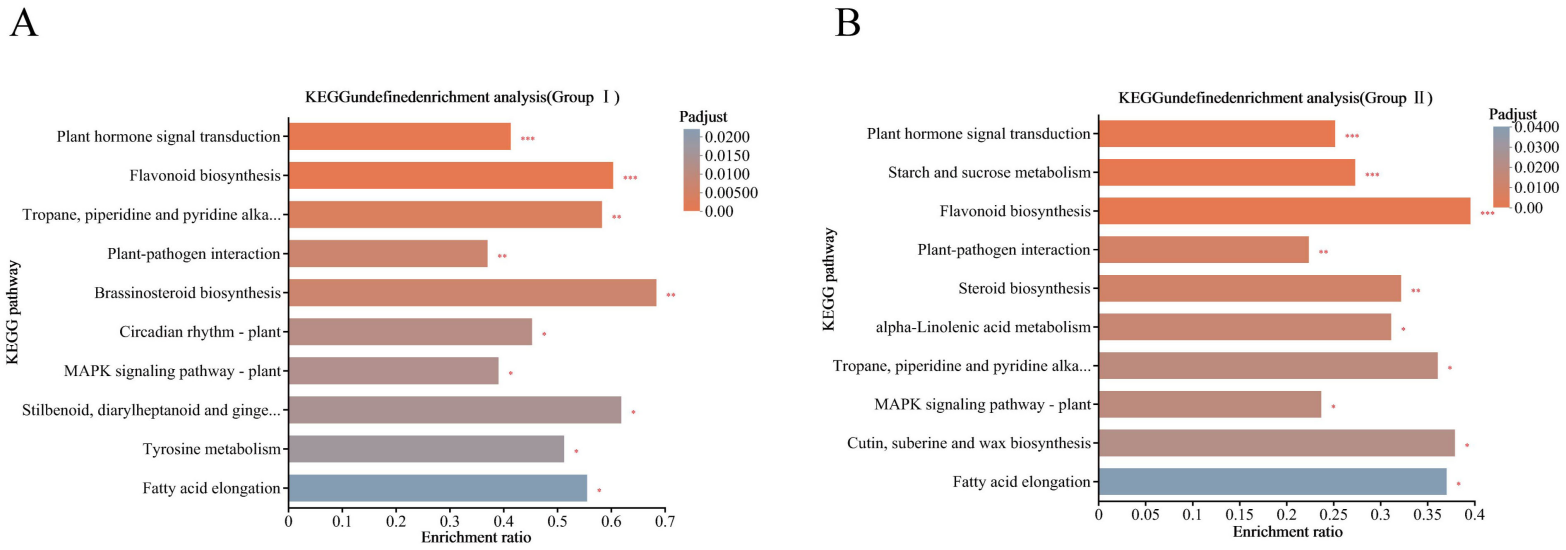
KEGG pathway enrichment analysis of DEGs under cold stress. **(A)** Group II DEGs; **(B)** Group II DEGs. Column color gradient indicates the significance of enrichment, where P< 0.001 is marked as ***, P< 0.01 is marked as **, and P< 0.05 is marked as *.

KEGG pathway enrichment analysis was further performed on the DEGs of the two comparison groups, Group I and Group II, in which the DEGs of Group I were mainly enriched in the phytohormone signaling, tropane, piperidine and pyridine alkaloid biosynthesis, flavonoid biosynthesis pathways, and starch and sugar metabolism pathways (**Figure 5A**), and the DEGs of Group II were mainly highlighted in phytohormone signaling pathway, starch and sugar metabolic pathways and MAPK signaling pathways (**Figure 5B**).

### 3.5 Weighted gene co-expression network analysis

**Figure 6:**
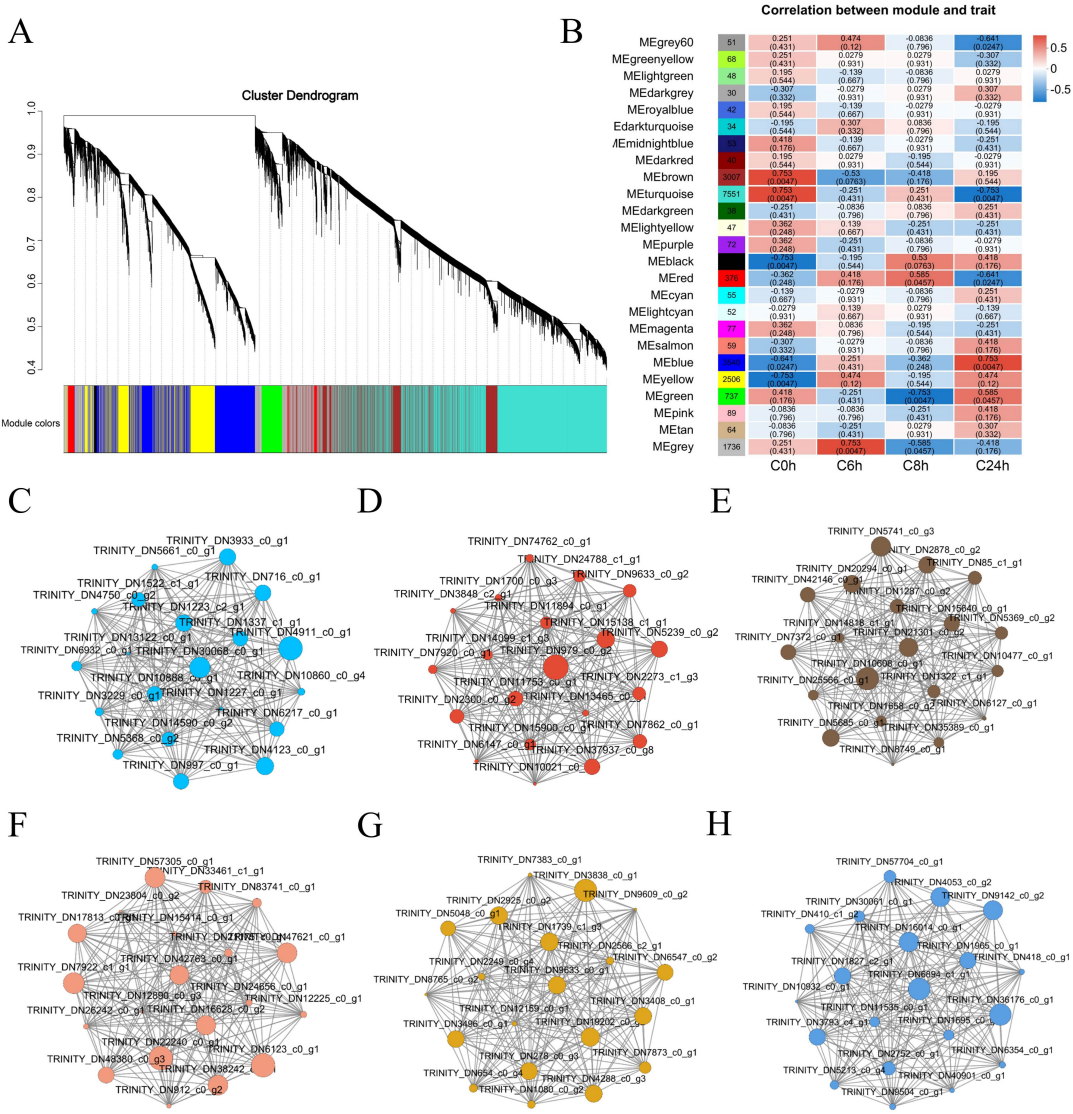
WGCNA of cold stress-related genes in *A catechu*. **(A)** Module division of gene expression trends, where a dendrite represents a gene and a color represents a module. **(B)** Correlation between modules and samples. The horizontal coordinates represent different samples, and the vertical coordinates represent different modules. **(C-H)** Six different modules, each containing one core gene, were screened in the co-expression network. These core genes may be core genes associated with cold stress in A *catechu*. **(A)** TRINITY_DN30068_c0_g1 (*ZMYND15*) **(B)** TRINITY_DN979_c0_g2 (*ABHD17B*) **(C)** TRINITY_DN21301_c0_g2 (*ATL8*) **(D)** TRINITY_DN42763_c0_g1 (*WNK5*) **(E)** TRINITY_DN9633_c0_g1 (*XTH3*) **(F)** TRINITY_DN6894_c1_g1 (*TPS*).

To identify potential genes for cold tolerance in *A. catechu*, a pre-processing of the data for 125,046 genes was first conducted. After this, the genes exhibiting low expression levels or insignificant coefficients of variation were filtered out. This process ultimately led to the identification of 20,474 genes. Subsequently, we performed WGCNA using these 20,474 genes and constructed a co-expression network associated with cold stress in *A. catechu* (soft power = 9). A total of 25 modules were detected in this analysis (**Figure 6A**). The correlation analysis between different modules and samples showed significant positive correlations between brown, turquoise, red, and blue (P<0.05, **Figure 6B**). After that, we further filtered these 25 modules. We selected 6 expression modules with high correlation with the sample, in which usually the greater the node connectivity, the more important. Based on this, we screened TRINITY_DN30068_c0_g1 (*ZMYND15*), TRINITY_DN979_c0_g2 (*ABHD17B*), TRINITY_DN21301_c0_g2 *(ATL8*), and TRINITY_DN42763_c0_g1 (*WNK5*), TRINITY_DN9633_c0_g1 (*XTH3*) and TRINITY_DN6894_c1_g1 (*TPS*), which are the six genes as the key hub genes for cold tolerance in *A. catechu* (**Figures 6C-H**).

### 3.6 Quantitative real-time PCR of expression patterns of hub genes

**Figure 7:**
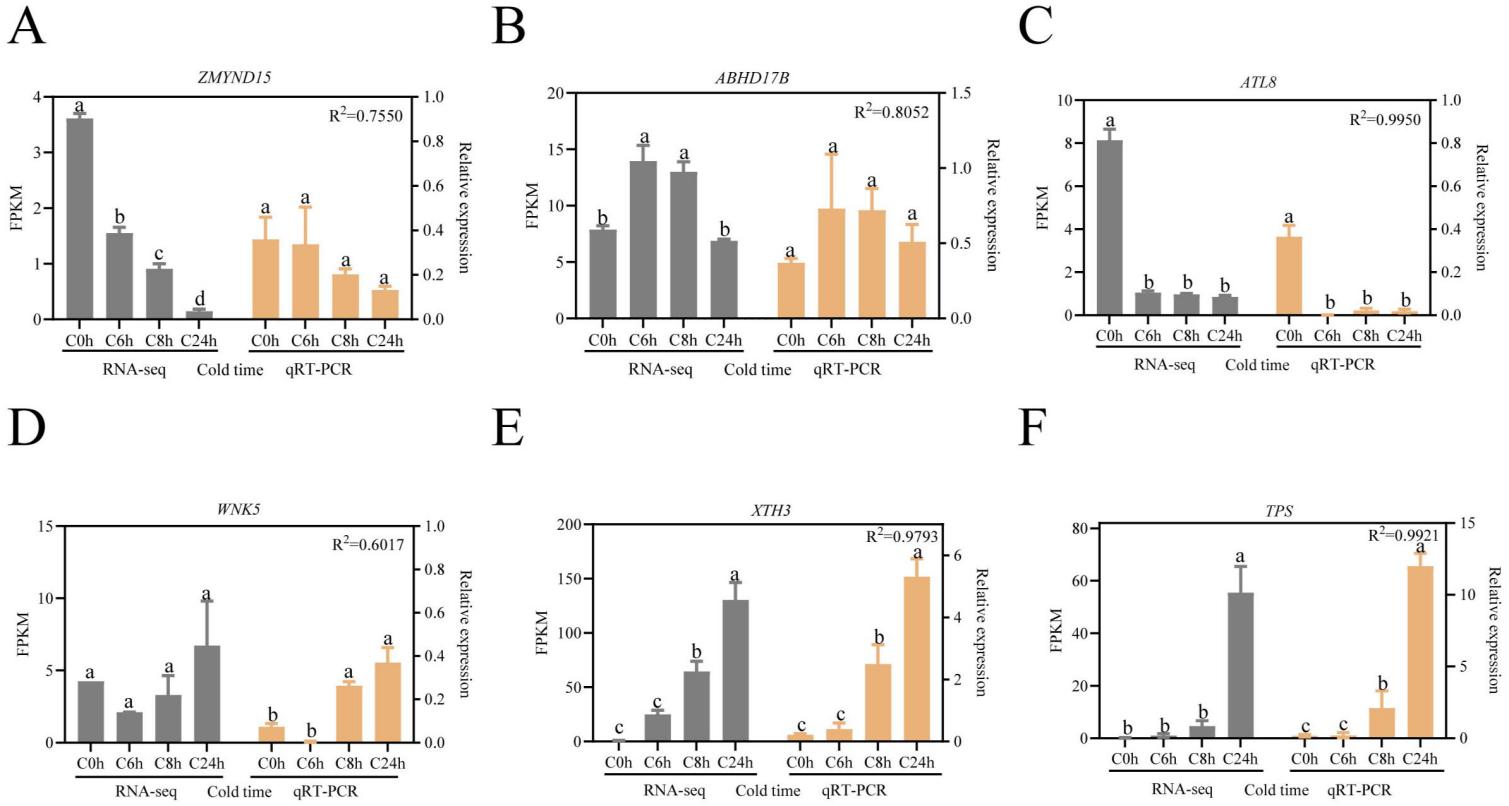
RT-qPCR verified the expression pattern of key hub genes. Data were analyzed using Tukey’s multiple comparison test at a significance level of P < 0.05, and error lines indicate standard errors. **(A)** *ZMYND15*: MYND-containing zinc binding protein 15. **(B)** *ABHD17B*: Hydrolysis enzyme-containing structural domain protein 17B. **(C)** *ATL8*: E3 ubiquitin ligase. **(D)** *WNK5*: Serine/Threonine Protein Kinase. **(F)** *XTH3*: xyloglucan endotransglucosylase/hydrolase 3 **(E)** *TPS*, trehalose-phosphate synthase. Lowercase letters (a, b, c) indicate statistically homogeneous groups based on Tukey’s multiple comparison test (P<0.05). Groups sharing the same letter are not significantly different, while those with different letters show significant differences.

To further validation of the reliability of the RNA-seq data, we analyzed the expression patterns of the six hub genes that had been screened using reverse transcription quantitative PCR (**Figure 7**). The statistical results demonstrated that the mean value of R^2^ between the data sets was 0.8581, indicating that the relative expression of the six DEGs was consistent with the corresponding RNA-seq results, thereby confirming the reliability of the RNA-seq data obtained in this study. Hub gene expression analysis revealed divergent temporal patterns: *ZMYND15* and *ATL8* exhibited progressive downregulation throughout cold exposure (**Figures 7A, C**), whereas *XTH3* and *TPS* demonstrated sustained upregulation (**Figures 7E, F**). *ABHD17B* demonstrated transient upregulation peaking at 6 hours, followed by decline (**Figure 7E**), which contrasts with the initial suppression followed by subsequent activation of *WNK5* (**Figure 7D**). These distinct temporal dynamics suggest functional diversification during the process of cold adaptation.

## 4 Discussion

### 4.1 Analysis of the physiological response of *A. catechu* under cold stress

**Figure 8:**
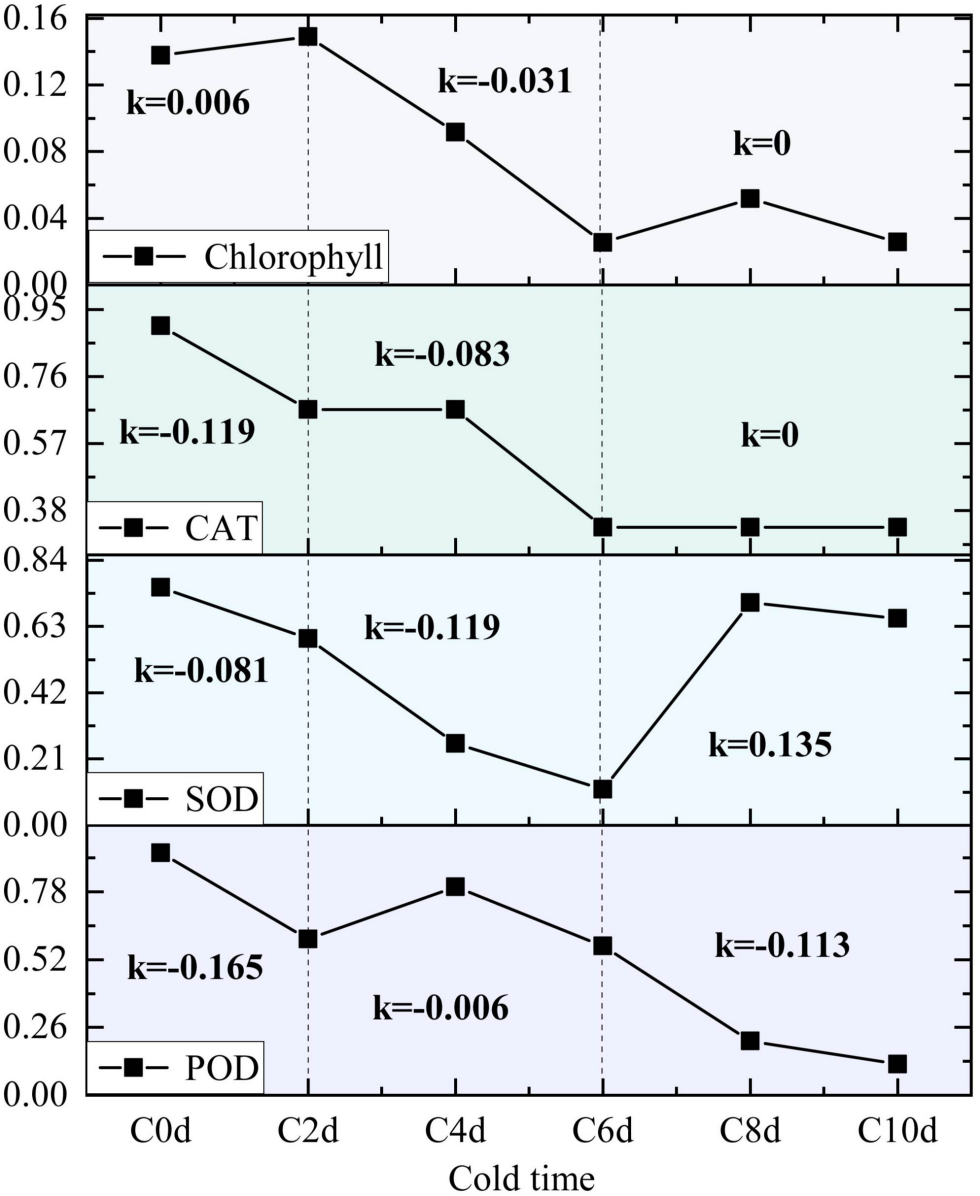
Line plots of the parameters of interest (POD, SOD, CAT, and Chlorophyll) as a function of stress time are presented, with the slope K representing the gradient between 0-2days, 2-6days, and 6-10days values.

Cold adaptation in plants is a multifaceted process of which the hallmarks are structural remodeling, antioxidant activation, and osmolyte dynamics (Soualiou et al., 2022). From a mechanistic perspective, cold exposure has been demonstrated to induce ultrastructural changes, including vesicle membrane destabilization. Furthermore, membrane integrity is structurally compromised by electrolyte leakage (Browse and Xin, 2001). However, plants have been observed to counteract oxidative stress by activating POD systems and regulating MDA levels to scavenge ROS. Moreover, they have been shown to maintain osmotic homeostasis via proline accumulation and soluble protein/sugar modulation, thereby preserving membrane integrity under cold stress (Alam et al., 2022; Kong et al., 2024). However, given the divergent physiological structures and metabolic mechanisms exhibited by different organisms, discrepancies in their temperature tolerance are to be expected. In the present study, the rate of change after cold stress was analyzed by calculating the slope of physiological indices of *A. catechu* at different cold stress time nodes. The enzymes POD, SOD and CAT demonstrated a rapid response to cold stress, exhibiting significant differences by the second day of exposure (**Figure 8**). And the changes in their activities showed a similar pattern: a slight decrease in the initial stage, a subsequent increase, and a trend of decreasing again. This pattern of change suggests an imbalance between reactive ROS and the defense system in *A. catechu* during adaptation to the cold environment, leading to intracellular free radical production and destruction of the defense system (Kim et al., 2024). Changes in indicators related to the photosynthetic system, such as chlorophyll content, exhibited a marked deceleration in the early days of the stress period (K = 0.006), and demonstrated greater variability from day 2 to day 6 (K = -0.031) (**Figure 8**). The potential underlying cause of this phenomenon is hypothesized to be a ROS burst, which has been shown to induce oxidative stress, leading to chlorophyll catabolism and a consequent decrease in photosynthetic rate (Chen et al., 2022). This result was also corroborated in the phenotype of *A. catechu*, which exhibited slight yellowing of the leaves at day 6 of the cold stress.

Multivariate analysis (hierarchical clustering and OPLS-DA) delineated two cold-response phases in *A. catechu*: the first stage was from 0 days to 4 days, and the second stage was from 6 days to 10 days. This finding indicates that, in *A. catechu*, 0 days to 4 days may be indicative of the physiological state at the initial stage of cold stress, with a shift in physiological acclimatization from day 6, which is also consistent with the findings of the present study that most of the physiological indices were significantly changed at day 6. Multivariate analysis also revealed key cold-tolerance regulators in *A. catechu*. PCA was able to identify a number of positive correlates, including SOD, POD, CAT, chlorophyll, H_2_O_2_ and proline. OPLS-DA, however, identified a different set of critical factors, namely POD (VIP = 1.85) and Chlorophyll (VIP = 1.76). At the beginning of cold stress, POD, as an oxidoreductase, can be rapidly activated to maintain the homeostasis of free radical content in plants and safeguard cells’ normal physiological function and structural integrity (Jan et al., 2023). However, with the prolongation of the stress time, this defense mechanism is gradually affected, and the efficiency of carbon dioxide and photosynthetic product utilization decreases, reducing photosynthetic efficiency and affecting the normal growth of plants (Yoo et al., 2019). This series of change processes also suggests that *A. catechu* transitioned from an oxidative defense mechanism to a photosynthetic acclimation mechanism during cold acclimation.

### 4.2 Identification of genes for cold stress-related metabolic pathways in *A. catechu*

Transcriptome analysis is an important tool for decoding plants’ spatiotemporal gene regulatory network of abiotic stress (Gong et al., 2020; Kou et al., 2018). This study designed DEGs as two independent groups to assess the comprehensive changes in gene expression in *A. catechu* under cold stress. The first group involved the comparison of the control group (C0d) with each stress duration, with the aim of capturing the gene expression dynamics during the initial response phase. The second group analyzed the differential expression among samples with different stress durations after excluding the control group to reveal the adaptive changes of genes over time progression. This approach provides a multidimensional perspective that facilitates an in-depth understanding of the molecular response mechanisms of *A. catechu* to cold stress. The results demonstrated a 3.1-fold increase in the number of DEGs from 6 to 24 hours, indicating that the transcriptome response of *A. catechu* underwent significant changes with the progression of the stress process.

**Figure 9:**
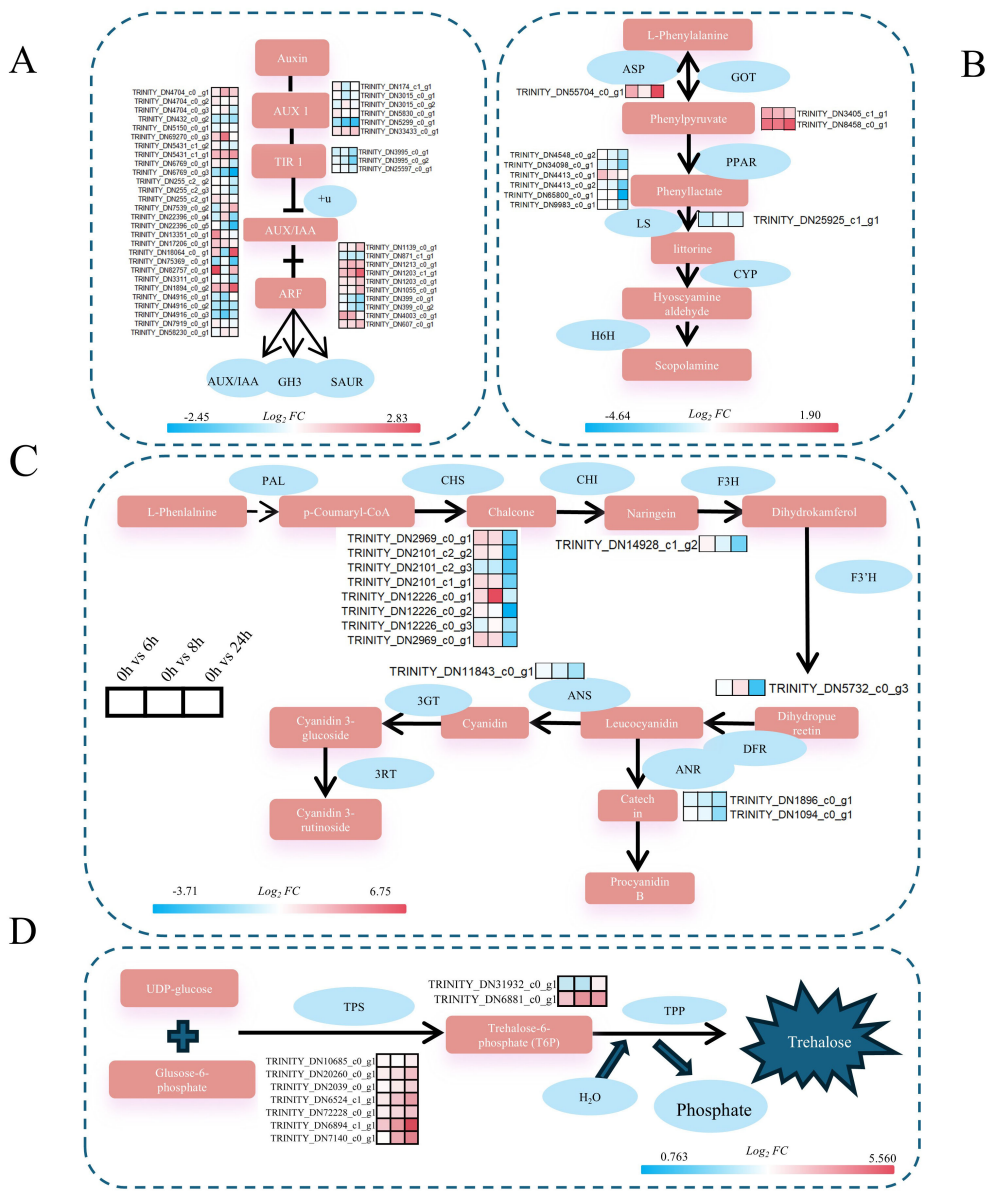
Gene expression profiles related to key synthetic pathways of cold stress in *A catechu*. The numbers in the legend indicate the *log_2_FC* value of the gene after treatment compared to the control (C0h). **(A)** Expression profiles of key genes in the growth hormone signaling pathway. **(B)** Expression profiles of key genes of the alkaloid synthesis pathway, *GOT*, *ASP*, *LS*, and *PPAR*. **(C)** Expression profiles of key genes in the flavonoid biosynthetic pathway. **(D)** Expression profiles of key genes in the alginate synthesis pathway. **(A)** *AUX1*, Auxin-Resistant 1; *TIR1:* Transport inhibitor response protein 1; AUX/IAA, growth hormone primary response genes; *ARF*, Auxin Response Factors; *GH3*, growth hormone amide synthase gene; *SAUR*, growth hormone up-regulated small RNA gene. **(B)** *ASP*, Asparagine synthetase; *GOT*, Aspartate aminotransferase; *PPAR*, Peroxisome Proliferator-Activated Receptor; LS, Littorina Synthase; *CYP*, enzyme tropine synthase; *H6H*, ranolazine 6B-hydroxylase. **(C)** *PAL,* phenylalanine deaminase; *CHS*, chalcone synthase; *CHl*, chalcone isomerase; *F3H*, flavonol 3-hydroxylase; *F3’H*, flavonol 3’-hydroxylase; *DFR*, dihydroflavonol 4-reductase; *ANR*, erythrocyanidin reductase; *ANS*, anthocyanin synthase; *3GT*, flavonol 3-0-glucosyltransferase; *3RT*, cornflavonoid 3-rutinoside. **(D)** *TPS*, trehalose phosphate synthase; *T6P*, trehalose-6-phosphate.

After cold stress in *A. catechu*, enrichment analysis of DEGs revealed that they were mainly enriched in phytohormone signaling, tropane, piperidine, pyridine alkaloid biosynthesis, and flavonoid biosynthesis pathways. In phytohormone signaling, we identified the *ARF* transcription factor. *ARF* can not only regulate the activity of target genes by recognizing specific sequences in promoter regions, thus adjusting cold metabolic pathways in plants and improving their adaptation to cold stress, but also regulate endosomal transport and growth hormone efflux in direct response to cold stress signals (Shani et al., 2017; Tolosa and Zhang, 2020). In this study, *ARF7* (TRINITY_DN1203_c1_g1) was significantly up-regulated under cold stress (**Figure 9A**). Nevertheless, in oil palm of the same genus, the expression level of *EgARF7* was observed to change in response to abiotic stresses such as drought and high salt. Therefore, it is speculated that *ARF7* is related to abiotic stress (Jin et al., 2022). Apart from this, the expression level of the *AUX/IAA* gene family member *IAA7* (TRINITY_DN1894_c0_g2) progressively increased with prolonged exposure to cold stress in *A. catechu*. The *AUX/IAA* gene regulate auxin signaling by interacting with auxin-responsive factors (ARFs), which regulate plant growth, development, and response to abiotic stresses (Zhang et al., 2021). Rice studies have demonstrated that the homologous gene *OsIAA20* mediates tolerance to drought and salt stress through the ABA pathway, and the functional significance of the family of genes in stress acclimation is evidenced by the reduced resistance of *OsIAA20* RNAi transgenic lines (Shani et al., 2017).

In addition to hormone signaling pathways, synthesizing secondary metabolites is critical in abiotic stress responses. In *A. catechu*, arecoline, a key bioactive compound, exhibits significant medicinal properties (Manimekalai et al., 2018). This study identified asparagine synthetase (*ASP*), a pivotal enzyme that catalysis the first step of arecoline biosynthesis through pyridine ring formation (Gaufichon et al., 2010). It is noteworthy that the expression of *ASP* was significantly increased under cold stress conditions, reaching a maximum at 24 hours of exposure (**Figure 9B**). Concurrently, flavonoids, another class of pharmacologically active compounds in *A. catechu* (Song Y. et al., 2022), contribute to cold tolerance by enhancing antioxidant capacity and ROS scavenging (Song F. et al., 2022; Zhao et al., 2024). In this study, *CHS1* (TRINITY_DN2969_c0_g1), a key enzyme in flavonoid biosynthesis (Peng et al., 2019), exhibited progressive down-regulation with prolonged stress duration (**Figure 9C**). In Arabidopsis, the cold-sensitive mutant *CHS1–2* exhibited no substantial deviation from the wild type at room temperature, but showed symptoms of growth arrest and leaf yellowing under low temperature conditions. This finding suggests that *CHS1* may plays a significant role in plant cold stress response (Wang et al., 2013).

In addition, the sugar metabolism pathway identified nine genes related to trehalose synthesis. In *A. catechu*, both *TPS* (TRINITY_DN6894_c1_g1, encoding trehalose phosphate synthase) and *T6P* (TRINITY_DN6881_c0_g1, an intermediate of trehalose synthesis) were consistently up-regulated with the increase of stress time (**Figure 9D**). From a mechanistic perspective, the impact of cold stress on plant biology involves the regulation of sucrose synthesis, degradation, and transport. This regulatory process is achieved through the activation of the *TPS* gene expression, which in turn modulates the level of cold tolerance exhibited by the plant. To illustrate this concept, consider the case study of wheat plants. In these plants, the genetic manipulation of *TaTPS11-6D* results in an increase in sucrose content within the leaves under low-temperature conditions. Concurrently, this manipulation leads to a decrease in the rate of sucrose degradation, with the sucrose being converted into glucose and fructose. Furthermore, this manipulation enhances the translocation of source sugars to the tiller nodes in the leaves (Lu et al., 2024). Furthermore, exogenous supplementation of *T6P*, a proposed precursor for trehalose biosynthesis, has been shown to mitigate temperature stress-induced physiological dysfunction and metabolic perturbations (Raza et al., 2024). Collectively, these findings advance contribute to our understanding of molecular adaptations in *A. catechu* under abiotic stress and inform targeted strategies for enhancing its stress resilience.

Beyond the core pathways discussed, our extended analysis of the MAPK signaling and brassinosteroid biosynthesis pathways (**Supplementary Figure S4**) revealed dynamic transcriptional reprogramming under cold stress. Expression pattern analysis showed that *MPK3* was persistently and significantly up-regulated upon exposure to cold stress. This suggests that MAPK signaling is activated in *A. catechu* in response to stress and may be involved in regulating downstream stress responses. In other studies, *MPK3* was found to inhibit the expression of *CBF* genes and reduce plant cold tolerance in plant cold stress mainly through negative regulation of the ICE1-CBF-COR signaling pathway (Zhao et al., 2017). For instance, *TaMPK3* overexpression in wheat was able to improve cold hardiness in Arabidopsis by enhancing the expression of *TaICE41* in the ICE1-CBF-COR signaling pathway (Wang et al., 2024). In addition, a total of six DEGs were identified in the Brassinosteroid Biosynthesis pathway. Gene expression patterns showed that the *TC4H* was significantly up-regulated and *BRI1* was significantly down-regulated at 24 hours of cold stress. These results suggest that the Brassinosteroid Biosynthesis pathway may play a role in cold stress, which is worthy of further study.

In addition to enzymatic and metabolic adaptations, transcriptional regulation has been identified as a key component of the cold stress response. Transcription factor (TF) analysis identified 809 cold stress response transcription factors, primarily from the MYB superfamily, AP2/ERF, bHLH, WRKY, and NAC families. Among these, the MYB superfamily contains the most significant transcription factors, with 150 members, accounting for 19%. Next is the AP2/ERF family, with 80 transcription factors (10%) (**Supplementary Figure S5**). Notably, both of these gene families are involved in the low-temperature resistance mechanism dependent on the “ICE-CBF-CRO” signaling pathway, suggesting that they may play a key role in the low-temperature response of *A. catechu*.

### 4.3 Identification of cold stress pivotal genes in *A. catechu*

In the present study, we employed WGCNA to systematically analysis transcriptome data, thereby revealing critical cold-responsive genes in *A. catechu*. These candidate genes elucidate molecular regulatory networks underlying the species’ adaptation to low-temperature stress. The pivotal gene *ATL8*, a plant-specific E3 ubiquitin ligase (Luo et al., 2019), exhibited a 19.21-fold downregulation at 24 hours of cold exposure in this study, thus highlighting ubiquitination as a central regulatory mechanism in cold acclimation. In essence, the function of *ATL8* is to negatively regulate cold responses in rice, achieved by targeting *OsGF14d* homologs for proteasomal degradation (Cui et al., 2022). Intriguingly, this ubiquitination-mediated suppression may activate compensatory phosphorylation cascades: *WNK* kinases (serine/threonine kinases) showed specific induction at 24 hours of cold stress in *A. catechu*, mirroring *AcWNK8*/*AcWNK12* upregulation observed in Acorus calamus under 4°C treatment (Ji et al., 2023). From a mechanistic perspective, *WNK* kinases have been hypothesized to function as messengers, relaying ROS signals through MAPKKK phosphorylation. This, in turn, has been shown to trigger a series of downstream pathways, including Nrf2-ARE signaling and heat shock protein activation. Collectively, these pathways have been demonstrated to regulate antioxidant gene expression (Chen et al., 2021; Son et al., 2011).

Concurrently, cell wall remodeling via *XTH3* (xyloglucan endotransglucosylase) (Takahashi et al., 2019), enhanced mechanical strength through lignin-xyloglucan network restructuring, a known adaptation to improve cold tolerance (Rose et al., 2002; Tan et al., 2024). Furthermore, *TPS* (trehalose-6-phosphate synthase) displayed progressive upregulation during prolonged cold stress, paralleling *TPS9* induction in cold-stressed peanut (Zhong et al., 2024). This expression dynamics likely reflects *TPS* dual functionality: (1) trehalose accumulation as an Osmo protectant, and (2) signaling molecule roles in stress response modulation (Xu et al., 2017). *TPS9*-mediated photosystem II protective mechanisms activation may underpin cellular homeostasis maintenance under cold stress.

Among the six hub genes, the *TPS* gene plays a pivotal role in plant cold tolerance and metabolic regulation (Gao et al., 2021). Consequently, we conducted a targeted phylogenetic analysis of the WGCNA-identified *TPS* hub genes by constructing a neighbors-joining tree that includes orthologs from rice and Arabidopsis (Vandesteene et al., 2012; Yang et al., 2012). The analysis revealed that the 11 *TPS* genes were clustered into two distinct branches (Branch A/B; **Supplementary Table S7**). Specifically, Branch A contains two Class II *TPS* genes (*AcTPS6*, *AcTPS7*) from the B1 subfamily, while Branch B includes three Class I genes (*AcTPS1–3*) from the A3 subfamily. Due to the distant evolutionary relationship of *A. catechu*, six *TPS* genes formed a separate branch (**Supplementary Figure S6**), indicating potential cold-response mechanisms that are distinct from those of other species.

### 4.4 Different low-temperature response pathways in *A. catechu*

**Figure 10:**
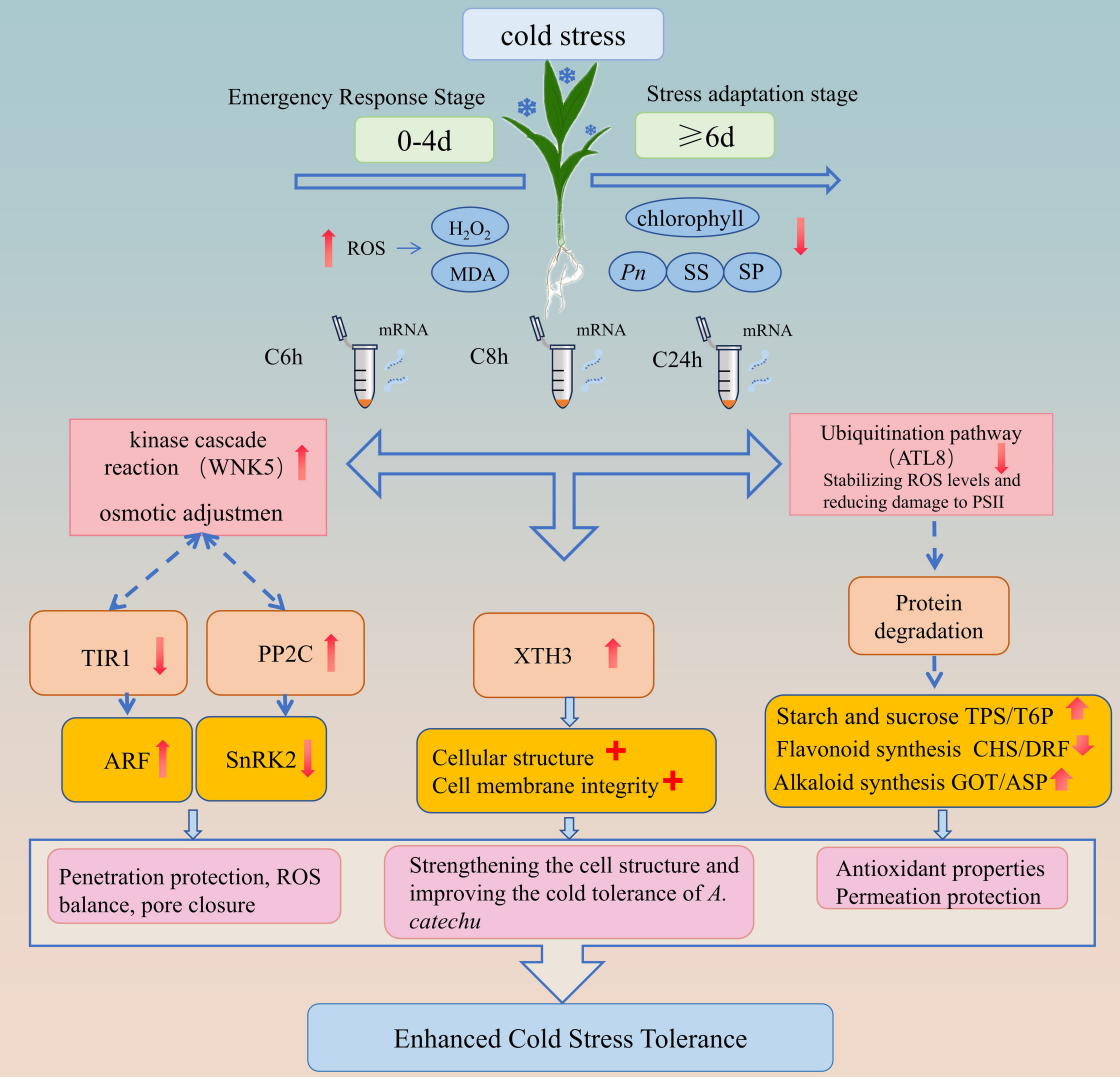
Proposed cold adaptation network for *A. catechu*. Dashed lines indicate inferred interactions.

Based on the results of this study, we have integrated a comprehensive pathway for cold hardiness in *A. catechu* (**Figure 10**). The model describes the various physiological and molecular responses of *A. catechu* to cold stress: physiological dynamics show an emergency phase (0–4 days) characterized by ROS accumulation (H_2_O_2_/MDA) and a late acclimatization phase (>6 days) characterized by chlorophyll stabilization and peroxidase (POD) activation.

At the molecular level, our findings indicate that cold adaptation in *A. catechu* may be facilitated by three core mechanisms. The primary mechanism of the first pathway involves *WNK* kinase which enhances cold tolerance by modulating the signaling cascades of the hormones IAA and ABA (Manuka et al., 2018; Saddhe et al., 2021). Evidence from other plant studies (Sah et al., 2016; Waadt et al., 2019; Xie et al., 2014), has demonstrated that *WNK* positively regulate the ABA signaling pathway in response to abiotic stress. In *A. catechu*, cold stress may induce signal transduction through cellular events such as alterations in membrane fluidity, activating calcium ion channels, or inducing ROS production, ultimately converging on *WNK* kinase. Activated *WNK* directly phosphorylates and enhances *SnRK2* kinase activity (**Supplementary Figure S7**), thereby initiating the ABA signaling cascade in response to cold stress. Furthermore, *WNK* may mediate the degradation of *AUX/IAA* repressor proteins by responding to changes in IAA expression, thus releasing *ARF* transcription factors to engage in stress adaptation. As a signaling hub, *WNK* modulates critical nodes in multiple hormone pathways (particularly *SnRK2* in the ABA pathway) through phosphorylation, synergistically regulating the interaction between IAA and ABA signaling pathways. This collectively activates the expression of stress response genes, ultimately coordinating physiological adaptive responses such as the accumulation of Osmo protective substances, maintenance of ROS homeostasis, and stomatal closure, significantly enhancing the cold stress tolerance of *A. catechu.*

The second pathway reveals the central role of *XTH* (xylose transglucosylase/hydrogenase) in cold stress responses: when plants encounter low-temperature stress, *XTH* expression is significantly upregulated, acting through multiple mechanisms to enhance stress resistance. These include synergistically regulating cellular osmotic homeostasis (Zhang et al., 2022), reorganizing cell wall structure to enhance extensibility and plasticity (Takahashi et al., 2021), and promoting the deposition of structural materials such as cellulose to enhance mechanical strength (Ma et al., 2024). In *A. catechu*, as cold stress duration increases, *XTH3* gene expression continues to be up-regulated, potentially through dynamically remodeling cell wall architecture to effectively mitigate cold-induced cellular damage, thereby enhancing the plant’s overall cold tolerance.

The third pathway involves the multifunctional regulatory role of *ATL* proteins (E3 ubiquitin ligase subfamily) in plant stress responses. *ATL* proteins are a subfamily of E3 ubiquitin ligases that are widely distributed in plants and play important roles in plant growth and development. Studies have shown that *ATL* can regulate the activity of enzymes such as chitinase (CHI), phenylalanine ammonia-lyase (PAL), polyphenol oxidase (PPO), catalase (CAT), peroxidase (POD), and superoxide dismutase (SOD) to regulate plant resistance (Lin et al., 2024). Additionally, *ATL* can help plants resist stress by enhancing the scavenging capacity of ROS and alleviating damage to photosystem II (PSII) (Song Q. et al., 2022). The *ATL* gene family also plays a key role in plant immune responses by regulating protein degradation to control immune signaling pathways, thereby influencing the transcription of secondary metabolites (Deng et al., 2017; Sierra-Cacho et al., 2025). In *A. catechu*, downregulation of *ATL* proteins may lead to protein degradation, degradation of transcription repressors, and release of transcription factors such as MYB and WRKY, thereby activating genes involved in the synthesis of alkaloids and flavonoids, enhancing the antioxidant capacity of *A. catechu* to cope with cold stress.

This framework integrates key pathways associated with cold tolerance in *A. catechu*. Based on transcriptomic co-expression patterns (WGCMA) and functional enrichment analysis, we propose a hypothetical regulatory network (**Figure 10**) that synthesizes these mechanisms. However, as the precise functions of central components (e.g., *ATL8*, *WNK5*) remain unvalidated in this species, the model represents a testable hypothesis requiring experimental verification.

## 5 Conclusion

This study systematically characterized cold stress responses in *A. catechu* through integrated phenotypic, physiological, and transcriptomic analyses. Key findings include: Reactive oxygen species (ROS) accumulation emerged as the primary physiological response within 24 hours of cold exposure (10°C). Multivariate analysis delineated two distinct phases: an early stress phase (0–4 days) marked by ROS surge, followed by an adaptive transition phase (≥6 days) characterized by POD activation and chlorophyll stabilization, identified as critical cold-hardiness biomarkers via OPLS-DA. Transcriptomic enrichment revealed cold-induced reprogramming of phytohormone signaling, alkaloid biosynthesis (tropane/piperidine/pyridine), and flavonoid pathways. WGCNA further identified three core modules: Ubiquitination-mediated protein turnover (*ATL8* hub gene), Phosphorylation cascades (serine/threonine kinases, e.g., *WNK5*), and Trehalose-6-phosphate metabolism (*TPS*-driven osmotic adjustment). Network centrality analysis prioritized *ZMYND15* (chromatin remodeling), *ABHD17B* (lipid signaling), and *XTH3* (cell wall remodeling) as master regulators of cold adaptation. These mechanistic insights establish a multi-tiered framework for understanding *A. catechu* cold tolerance, enabling targeted strategies.

## Data Availability

The data supporting this study’s findings has been deposited into the CNGB Sequence Archive (CNSA) (Guo et al., 2020) of the China National GeneBank Database (CNGBdb) (Chen et al., 2020) with access number CNP0007150.
