# Supplementary figures and images for: Deciphering the molecular signatures of tropical Areca catechu L. under cold stress: an integrated physiological and transcriptomic analysis
Source: Front Plant Sci. 2025 Jul 22;16:1624335. doi: 10.3389/fpls.2025.1624335 (PMC12321864; doi:10.3389/fpls.2025.1624335)

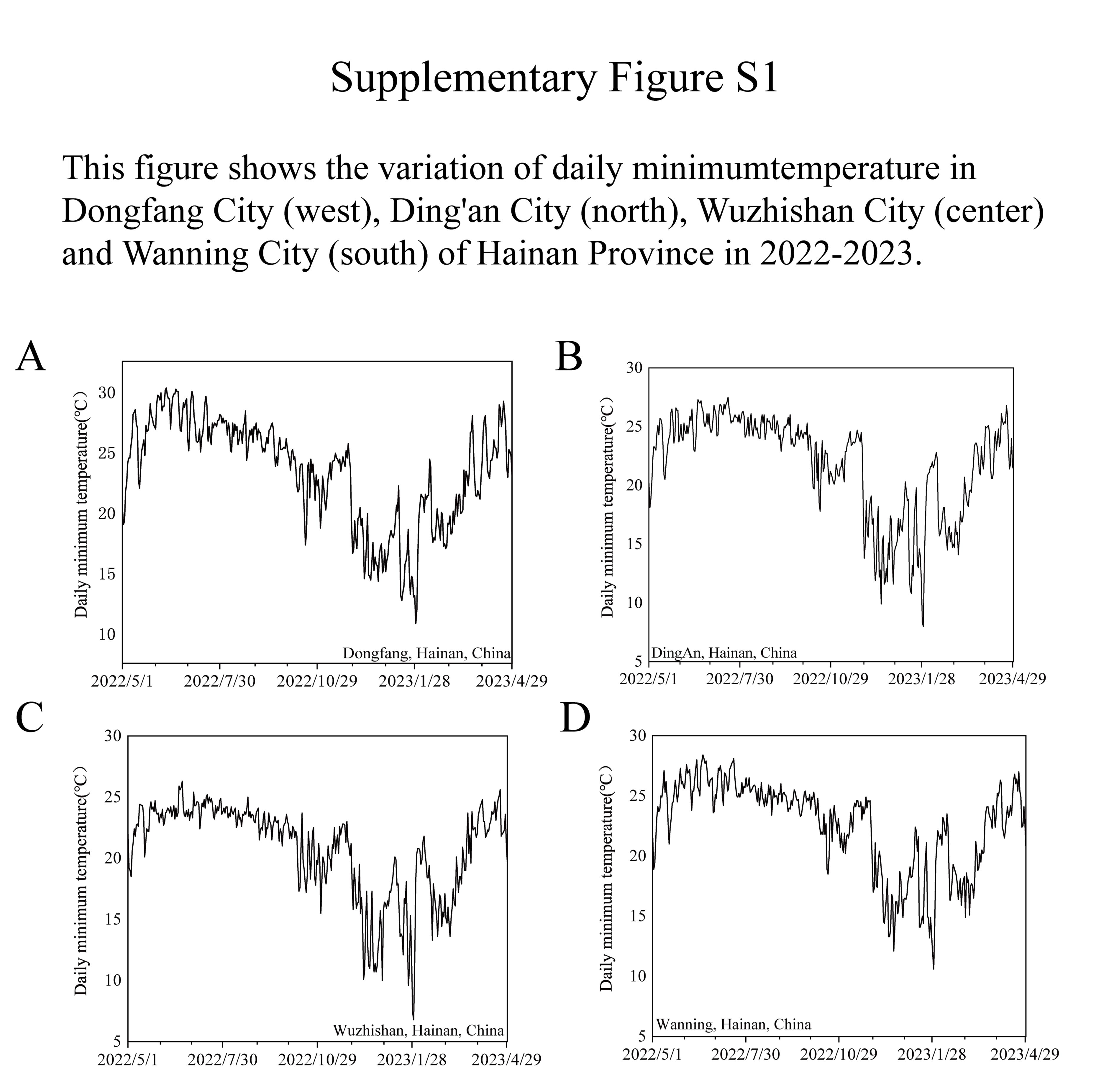

Supplement: Supplementary file 1 [file Image1.jpeg]

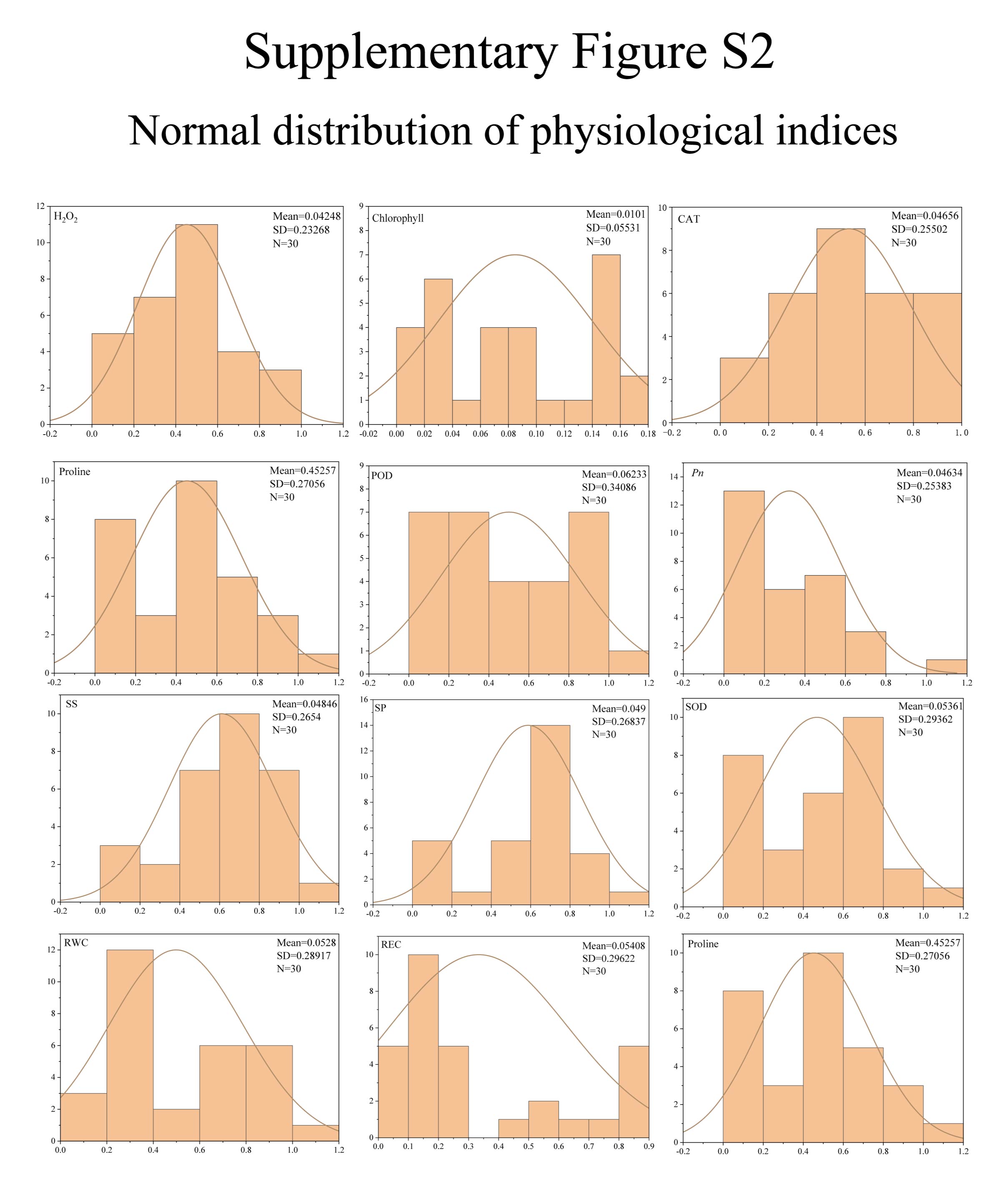

Supplement: Supplementary file 2 [file Image2.jpeg]

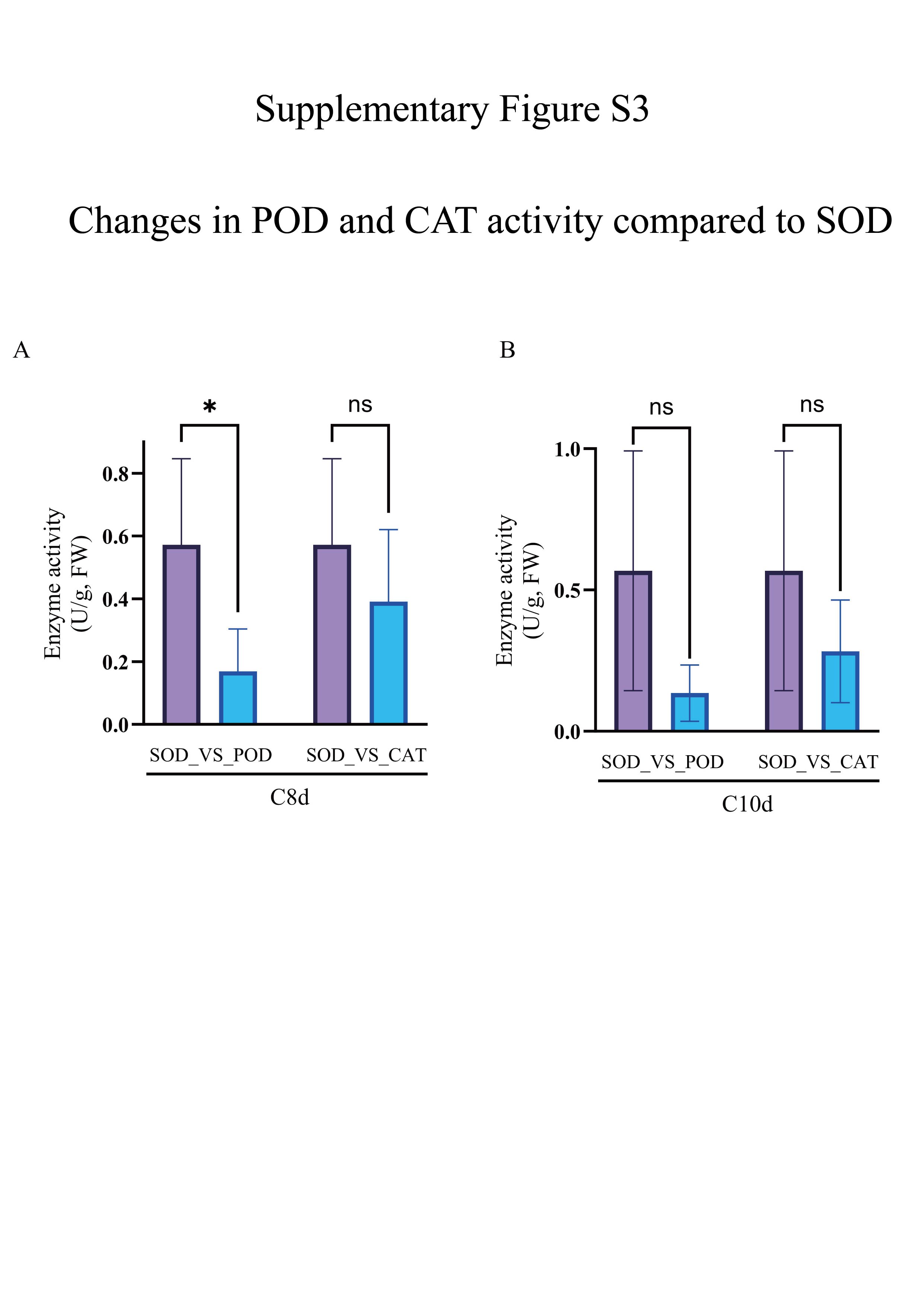

Supplement: Supplementary file 3 [file Image3.jpeg]

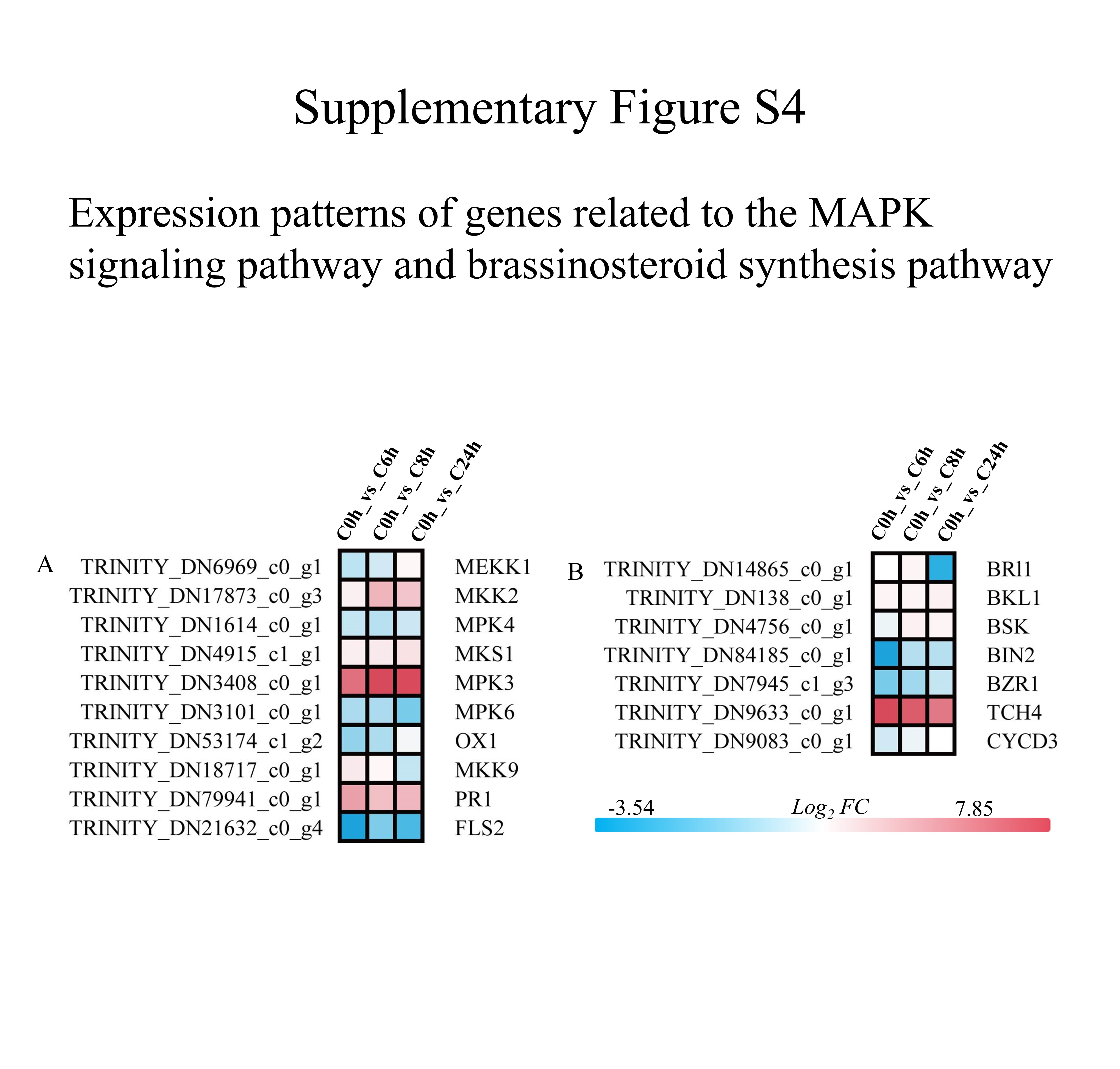

Supplement: Supplementary file 4 [file Image4.jpeg]

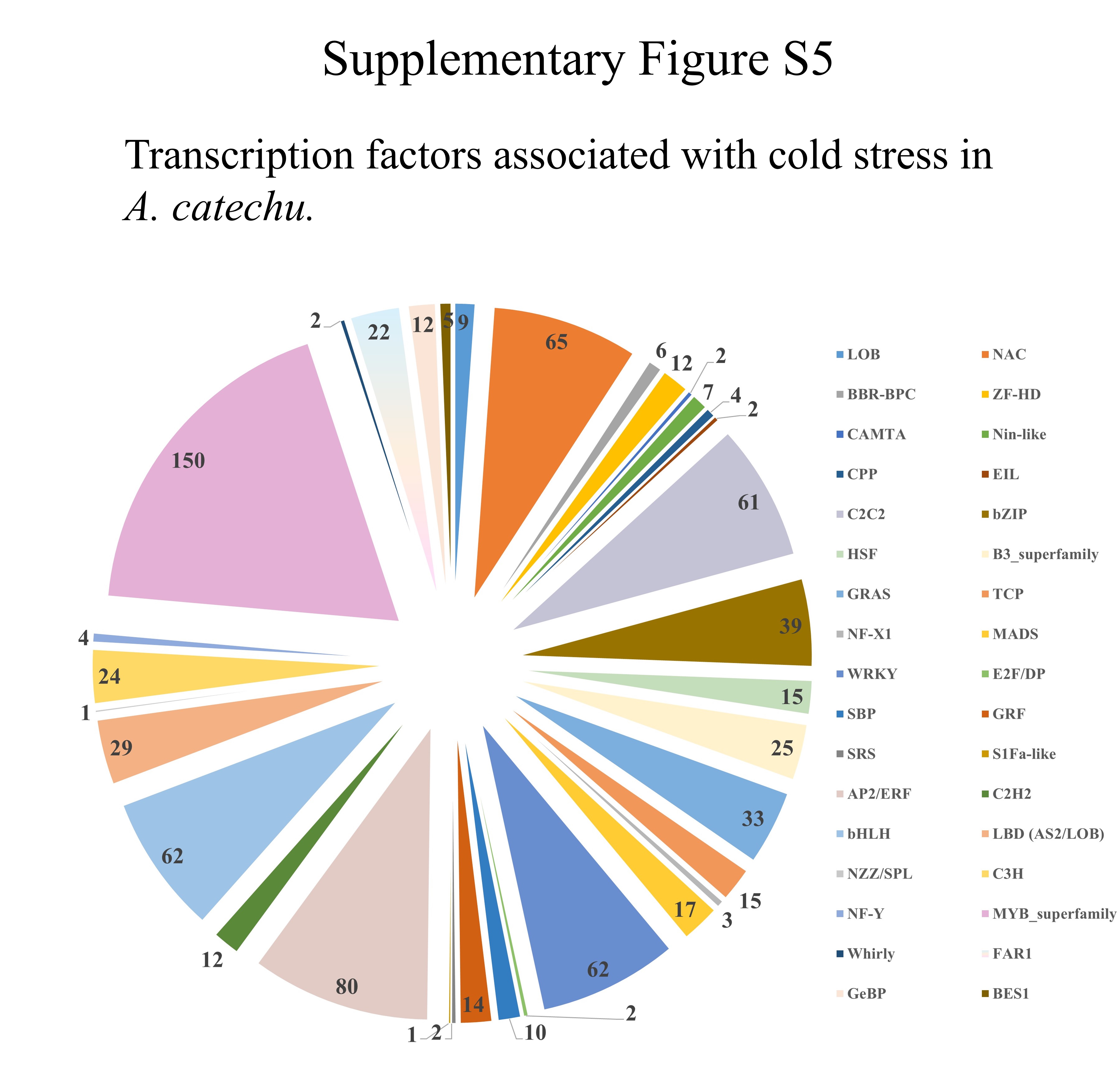

Supplement: Supplementary file 5 [file Image5.jpeg]

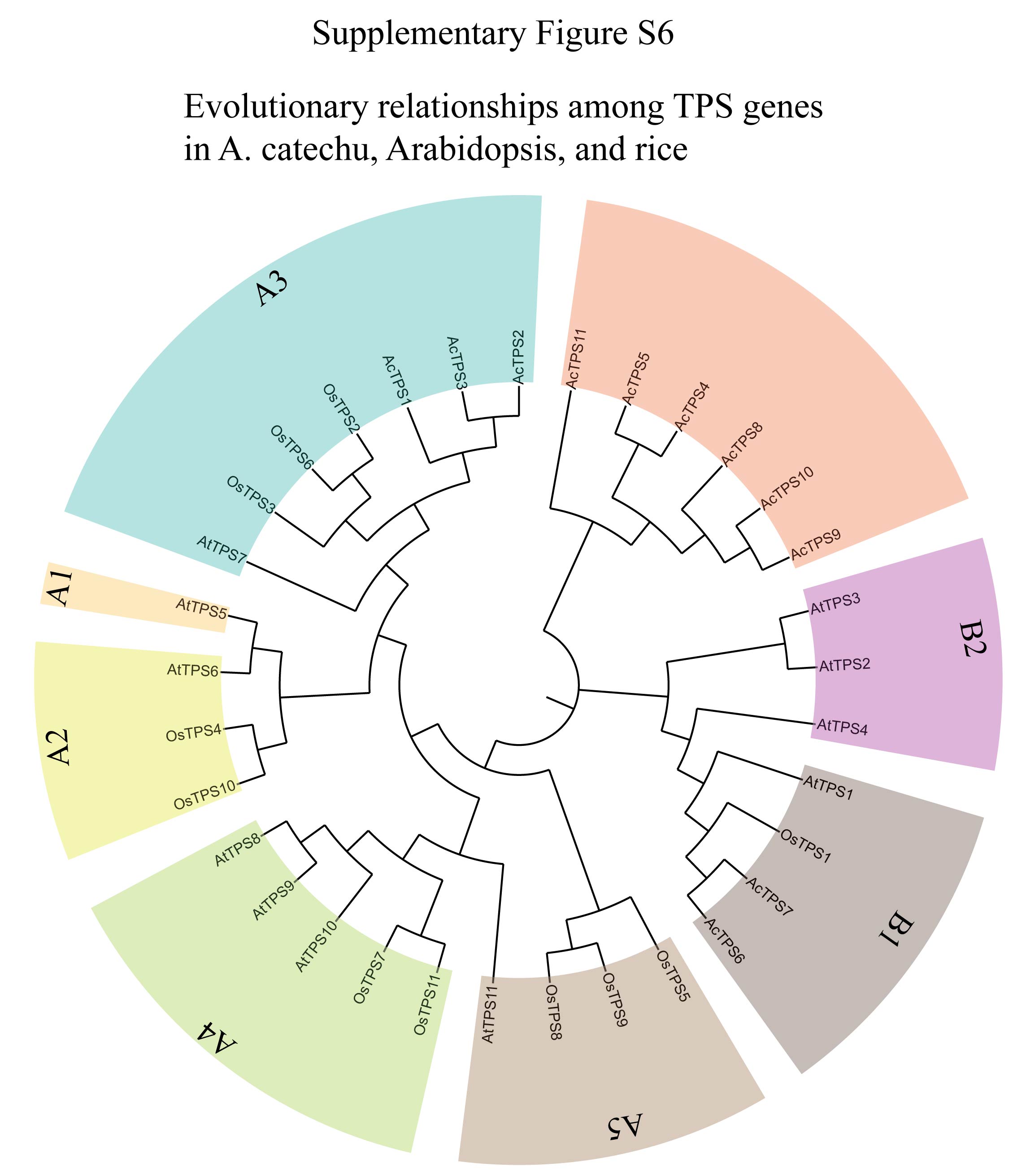

Supplement: Supplementary file 6 [file Image6.jpeg]

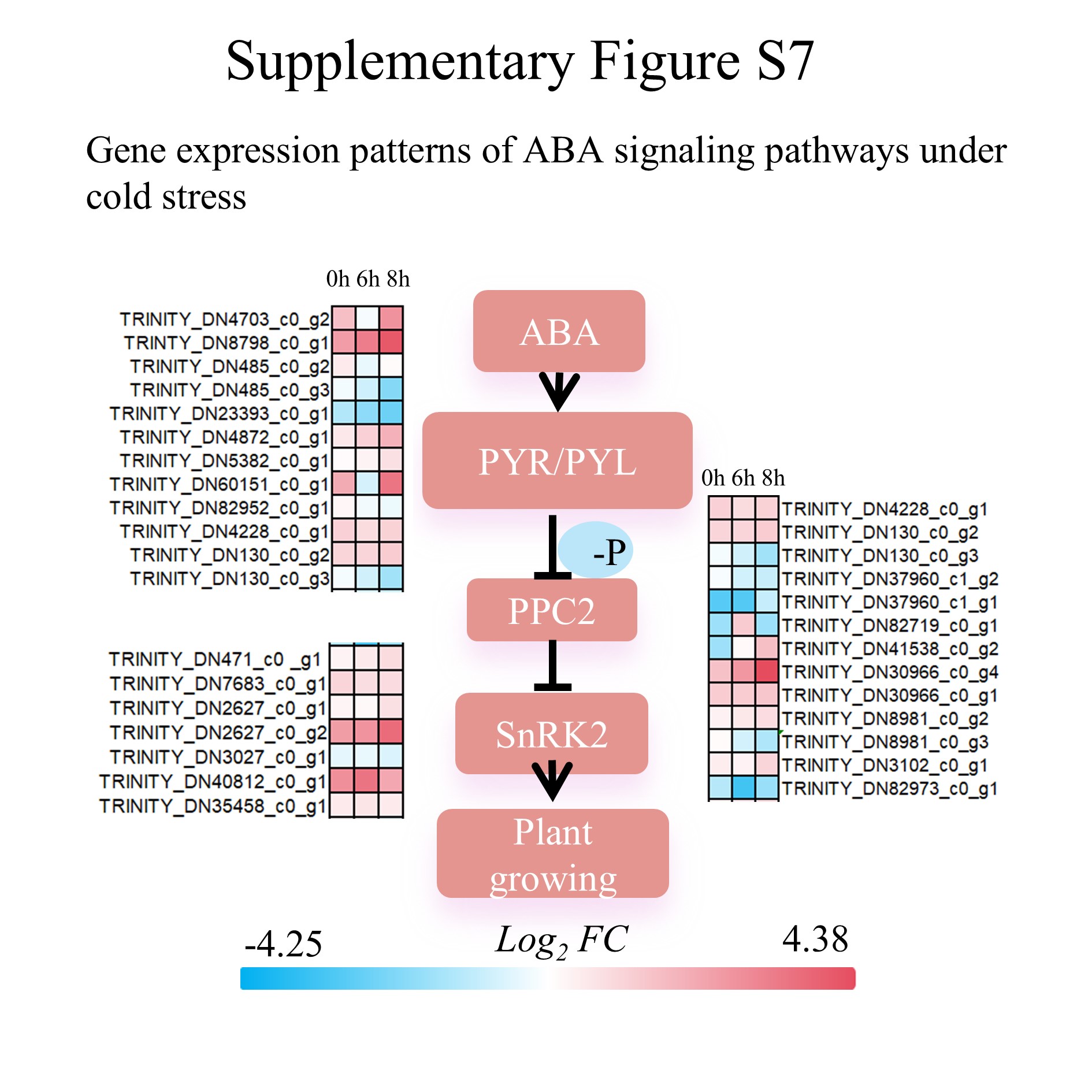

Supplement: Supplementary file 7 [file Image7.jpeg]
